# Male‐Biased Gut Microbiome and Metabolites Aggravate Colorectal Cancer Development

**DOI:** 10.1002/advs.202206238

**Published:** 2023-07-03

**Authors:** Ling Wang, Yi‐Xuan Tu, Lu Chen, Yuan Zhang, Xue‐Ling Pan, Shu‐Qiao Yang, Shuai‐Jie Zhang, Sheng‐Hui Li, Ke‐Chun Yu, Shuo Song, Hong‐Li Xu, Zhu‐Cheng Yin, Jun‐Qiu Yue, Qian‐Lin Ni, Tang Tang, Jiu‐Liang Zhang, Min Guo, Shuai Zhang, Fan Yao, Xin‐Jun Liang, Zhen‐Xia Chen

**Affiliations:** ^1^ Hubei Hongshan Laboratory Wuhan 430070 China; ^2^ Hubei Key Laboratory of Agricultural Bioinformatics College of Life Science and Technology Interdisciplinary Sciences Institute Huazhong Agricultural University Wuhan 430070 China; ^3^ Shenzhen Branch Guangdong Laboratory for Lingnan Modern Agriculture Genome Analysis Laboratory of the Ministry of Agriculture Agricultural Genomics Institute at Shenzhen Chinese Academy of Agricultural Sciences Shenzhen 518000 China; ^4^ Department of Medical Oncology Hubei Cancer Hospital Tongji Medical College Huazhong University of Science and Technology Wuhan 430079 China; ^5^ Wuhan Metwell Biotechnology Co., Ltd. Wuhan Wuhan 430075 China; ^6^ College of Food Science and Technology Huazhong Agricultural University Wuhan 430070 China; ^7^ Shenzhen Institute of Nutrition and Health Huazhong Agricultural University Shenzhen 518000 China; ^8^ College of Biomedicine and Health Huazhong Agricultural University Wuhan 430070 China

**Keywords:** colorectal cancer, fecal microbiota transplantation, gut microbiome, sexual dimorphism

## Abstract

Men demonstrate higher incidence and mortality rates of colorectal cancer (CRC) than women. This study aims to explain the potential causes of such sexual dimorphism in CRC from the perspective of sex‐biased gut microbiota and metabolites. The results show that sexual dimorphism in colorectal tumorigenesis is observed in both *Apc* ^Min/^ *^+^* mice and azoxymethane (AOM)/dextran sulfate sodium (DSS)‐treated mice with male mice have significantly larger and more tumors, accompanied by more impaired gut barrier function. Moreover, pseudo‐germ mice receiving fecal samples from male mice or patients show more severe intestinal barrier damage and higher level of inflammation. A significant change in gut microbiota composition is found with increased pathogenic bacteria *Akkermansia muciniphila* and deplets probiotic *Parabacteroides goldsteinii* in both male mice and pseudo‐germ mice receiving fecal sample from male mice. Sex‐biased gut metabolites in pseudo‐germ mice receiving fecal sample from CRC patients or CRC mice contribute to sex dimorphism in CRC tumorigenesis through glycerophospholipids metabolism pathway. Sexual dimorphism in tumorigenesis of CRC mouse models. In conclusion, the sex‐biased gut microbiome and metabolites contribute to sexual dimorphism in CRC. Modulating sex‐biased gut microbiota and metabolites could be a potential sex‐targeting therapeutic strategy of CRC.

## Graphical abstract

Sexual dimorphism in colorectal tumorigenesis also has been observed in CRC mouse models. The disparities in CRC incidence and mortality between the sexes may be attributed to sex‐biased gut microbiota and metabolites. Male‐biased gut metabolites aggravated colorectal tumorigenesis through the glycerophospholipid metabolism pathway. Adjusting these sex‐biased factors could potentially offer a targeted therapeutic approach for CRC.

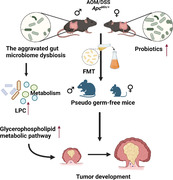

## 1 Introduction

Colorectal cancer (CRC) is the third most commonly diagnosed and second deadly cancer worldwide.^[^ ^1^ ^]^ It is noteworthy that more men than women are diagnosed and die from CRC each year.^[^ ^2^, ^3^ ^]^ Among CRC patients, men are diagnosed at an earlier age and on average 4–6 years younger at time of death than women.^[^ ^4^ ^]^ Such sex‐biased CRC survival has aroused wide attention because it could signify fundamental biological differences between men and women in cancer pathogenesis and response to therapy. However, CRC guidelines for screening or therapy have not taken sex‐biased recommendations into consideration. This might be due to the limited availability of sex‐biased preclinical data since most animal studies have used male mice for CRC induction to avoid possible influence of estrogen signaling.^[^ ^5^ ^]^ Therefore, revealing the mechanisms underlying sex differences with CRC animal models is important for the clinical implementation of precise medication.

Sex‐biased gut microbiome could potentially contribute to sex dimorphism of CRC development. CRC is a multifactorial disease, and several CRC risk factors have been identified such as lifestyle, genetic, and environmental factors.^[^ ^6^, ^7^, ^8^, ^9^ ^]^ Among them, the gut microbiome has been appreciated as an important player in CRC development. Gut microbiome significantly affects central nervous system (CNS) through the gut–brain axis transmitting the bidirectional biochemical signals between the gastrointestinal tract and the CNS.^[^ ^10^, ^11^ ^]^ The gut microbiota composition differs significantly between healthy people and CRC patients. In addition, the microbial diversity of healthy controls is significantly higher than that of the CRC patients.^[^ ^12^ ^]^ Compared to men, premenopausal women exhibited higher gut microbial diversity and higher abundances of multiple species with beneficial effects on host metabolism.^[^ ^13^ ^]^ However, the relationship between sex‐biased gut microbiome and sexual dimorphism in CRC remains largely unclear. Therefore, it is important to identify sex‐biased gut microbiome that affects differentially CRC formation in men and women. These sex‐biased gut microbiome might be valuable therapy targets and markers for cancer prognosis.

Various mouse models including genetically engineered mouse models (GEMM) and carcinogen‐induced models (CIM), have been developed in preclinical studies to recapitulate CRC in humans. *Apc* ^Min/^ *^+^* mouse model is a widely used GEMM to understand the molecular processes of familial adenomatous polyposis related CRC initiation and progression. While azoxymethane (AOM)/dextran sulfate sodium (DSS) induced CRC mouse model is more widely used to study inflammatory colorectal cancer.^[^ ^14^, ^15^ ^]^ Although sexual dimorphism has been demonstrated in CRC patients, it has not been reported in mouse models.^[^ ^13^ ^]^ In this study, we used these CRC mouse models to examine the sexual dimorphism of CRC, and evaluated the effect of sex‐biased gut microbiome on CRC progression. In addition, we found that the feces from male CRC mice or human patients promoted gut barrier dysfunction and intestinal inflammation in pseudo germ‐free mice, suggesting that sex‐biased gut microbiome contributed to CRC development. Modulating sex‐biased gut microbiota and metabolites could be a potential precise sex‐targeting therapeutic strategy for the prevention and treatment of sex‐biased CRC.

## 2 Results

### 2.1 Sexual Dimorphism in Tumorigenesis of CRC Mouse Models

In order to verify that CRC mouse models show similar sexual dimporism as CRC patients and thus could be used to study the sexual dimorphism in CRC patients, we used the transgenic CRC mouse model *Apc* ^Min/^ *^+^*, and compared the phenotypes between female and male *Apc* ^Min/^ *^+^* mice on standard diet with wildtype C57BL/6L mice used as control (Figure S1A, Supporting Information). As expected, females (260 days) survived 24 days longer than males on average with increase in mean survival time of 18.1% (Figure S1B, Supporting Information). Male *Apc* ^Min/^ *^+^* mice presented more and larger colorectal tumors than female *Apc* ^Min/^ *^+^* mice (Figure S1C, Supporting Information). Histological examination of colon sections indicated that male mice exhibited larger proportions of adenocarcinoma and high‐grade and low‐grade dysplasia than female mice (Figure S1D, Supporting Information). The colon sections of male mice displayed significantly more Ki‐67 positive cells than those of female mice, indicating the increased cancer cell proliferation in male mice (Figure S1E, Supporting Information). Goblet cells are specialized epithelial cells that are essential to the formation of the mucus barriers in the intestines.^[^ ^16^ ^]^ Periodic‐acid Schiff (PAS) staining of colon tissues showed that the average number of goblet cells in each crypt was smaller in male mice than in female mice (Figure S1F, Supporting Information). These data demonstrated the presence of similar sexual dimorphism in the CRC mouse model as in CRC patients.

**Figure 1:**
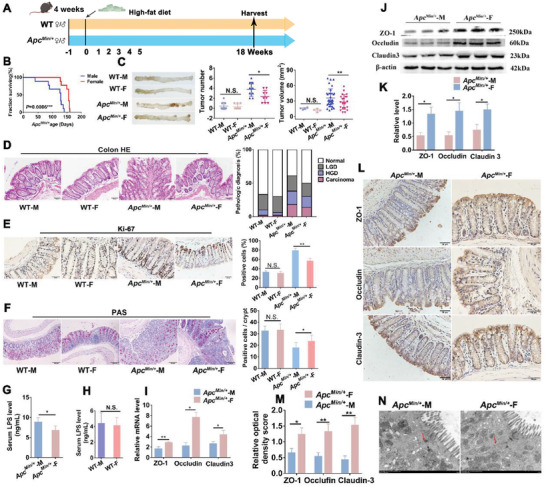
Sexual dimorphisms in the tumorigenesis of *Apc* ^Min/+^ mouse model with high‐fat diet. A) Experimental design for an *Apc* ^Min/+^ CRC mouse model and WT mice with high‐fat diet. B) Female had increased survival compared with male mice (*n* = 40 half male and half female). C) Representative image of colon at sacrifice. Tumor number and tumor volume in WT‐M, WT‐F, *Apc* ^Min/+^‐M, *Apc* ^Min/+^‐F mice (*n* = 9/each group). D) HE staining for pathologic diagnosis of mice colons. Quantitative analysis of pathologic score was calculated according to the following criteria: 0, normal; 1, LGD; 2, HGD; and 3, carcinoma. E) IHC staining for Ki‐67 of mice colons with quantitative analysis of Ki‐67 index. F) The number of colon goblet cells was evaluated by PAS staining. G) LPS concentration in serum of *Apc* ^Min/+^‐M and *Apc* ^Min/+^‐F mice. H) LPS concentration in serum of WT‐M and WT‐F mice. I) Expression level of gut barrier‐associated proteins ZO‐1, Occludin and Claudin‐3 in colon tissues of *Apc* ^Min/+^‐M and *Apc* ^Min/+^‐F mice using qRT‐PCR and J,K) Western blot with quantitative analysis. L,M) IHC for distribution of the adhesion molecule ZO‐1, Occludin and Claudin‐3 with quantitative analysis in colon tissues of *Apc* ^Min/+^‐M and *Apc* ^Min/+^‐F mice. N) Representative images of intercellular junctions of *Apc* ^Min/+^‐M and *Apc* ^Min/+^‐F mice by transmission electron microscope. IHC, immunochemistry; LGD, low‐grade dysplasia. Abx, antibiotics; HE, hematoxylin and eosin; HGD, high‐grade dysplasia. * *p* < .05, ** *p *< .01, N.S. no significant. Dot plot reflects data points from independent experiment.

Sex differences may relate to specific dietary pattern. To explore the sexual dimorphism during CRC development in different dietary patterns, we used *Apc* ^Min/^ *^+^* mouse model with high‐fat diet (**Figure** **1**A). Consistent with the results in the *Apc* ^Min/^ *^+^* model mice with standard diet, female mice (159 days) survived 39 days longer on average with an increase in mean survival time of 35.1% (Figure 1B). Meanwhile, male mice also presented more and bigger tumors (Figure 1C), larger proportion of adenocarcinoma and high‐grade and low‐grade dysplasia (Figure 1D), more Ki‐67 positive cells (Figure 1E), and less goblet cells (Figure 1F) than females, indicating that the sexual dimorphism in CRC was more obvious for *Apc* ^Min/^ *^+^* mice with high‐fat diet.

Furthermore, we used another CRC mouse model (AOM/DSS‐treated C57BL/6L mice) to determine whether the sexual dimorphism appeared only in specific CRC mouse models (Figure S2A, Supporting Information). Similarly, females (199 days) survived 44 days longer with an increase in mean survival time of 42% (Figure S2B, Supporting Information). Male mice also presented more severe symptoms than female mice (Figure S2C–F, Supporting Information), which was in line with the results in the *Apc* ^Min/^ *^+^* model mice, suggesting that the presence of sexual dimorphism in at least two mouse models.

All the results above indicated that CRC male mice presented more severe CRC symptoms than female mice, as reported in CRC patients, and thus the abovementioned CRC mouse models were applicable for further study on the mechanisms underlying the sexual dimorphism in CRC. Since transgenetic *Apc* ^Min/^ *^+^* mice underwent less treatment than AOM/DSS mice, and had more obvious sex dimorphism in CRC development in the case of high‐fat diet, we adopted *Apc* ^Min/^ *^+^* model with high‐fat diet to study the mechanisms underlying sexual dimorphism in CRC development.

### 2.2 Sex‐Biased Gut Microbiome Contributes to Sexual Dimorphism in CRC Development of Mice

The more severe CRC symptoms in male *Apc* ^Min/^ *^+^* mice might be resulted from their worse gut barrier function. To explore whether there was a difference in gut barrier function between male and female *Apc* ^Min/^ *^+^* mice, we examined the effect of sex on paracellular permeability in the colon of mice by measuring the serum lipopolysaccharides (LPS) level. The results showed that serum LPS concentration was elevated in male mice, compared with that in female mice (Figure 1G). Meanwhile, serum LPS concentration exhibited no significant difference between male and female WT mice (Figure 1H). Moreover, expressions of tight junction proteins (the key component of tight junction serving markers of gut barrier integrity) ZO‐1, Occludin, and Claudin‐3 were significantly reduced in male mice (Figure 1I–M). Consistently, the gut barrier structure under transmission electron microscopy showed the anabatic abnormalities of colonic intercellular junctions such as wider paracellular gap in male mice than in female mice, indicating that barrier function of male was more severely impaired (Figure 1N). Considering that gut barrier function was affected by gut microbiota, these results implied the involvement of gut microbiota in the sexual dimorphism in CRC tumorigenesis.

To evaluate the impacts of sex‐biased gut microbiome on sexual dimorphism in CRC tumorigenesis, we used an antibiotic cocktail to deplete the gut microbiota in male and female *Apc* ^Min/^ *^+^* mice. After *Apc* ^Min/^ *^+^* mouse antibiotic treatment, the difference of colorectal tumor number, volume difference, proportion of adenocarcinoma and grade dysplasia between male and female disappeared (Figure S3A–F, Supporting Information). We also performed the same experiment on AOM/DSS mice, and obtained similar results (Figure S3G–I, Supporting Information). These results suggested that gut microbiome might play an essential role in mediating sexual dimorphism in CRC development.

### 2.3 Gut Microbes from Male CRC Mice Contribute to Impaired Gut Barrier Function in Recipient Pseudo Germ‐Free Mice

**Figure 2:**
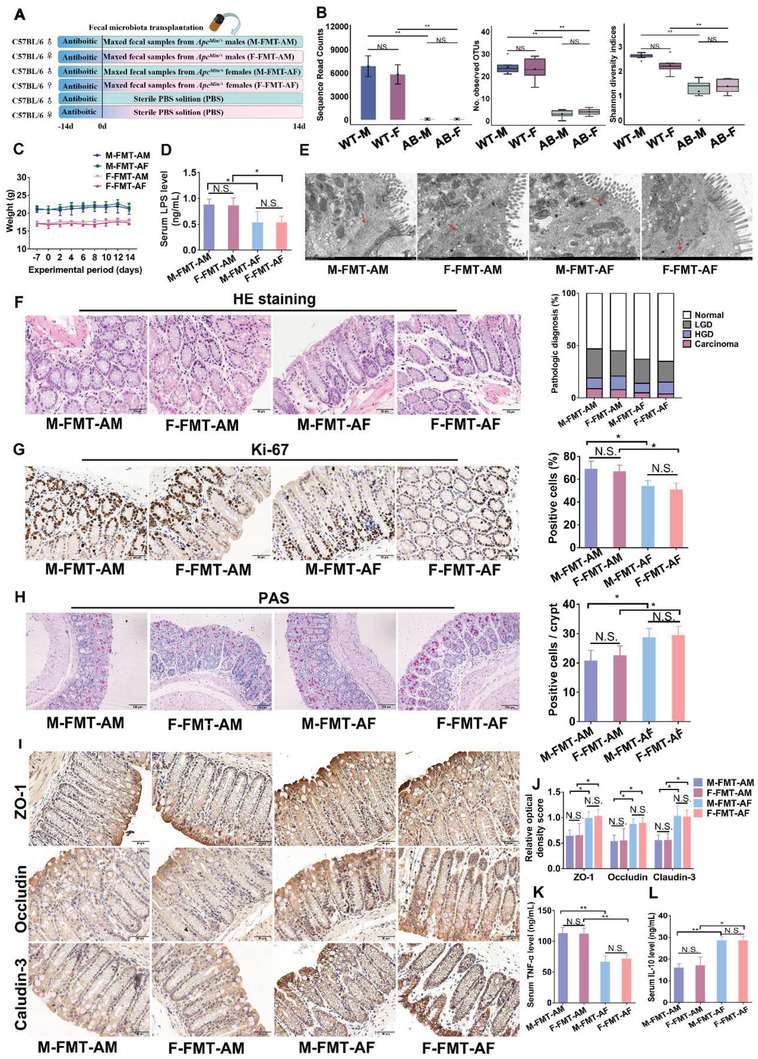
Gut microbes from male CRC mice contribute to more severe colorectal tumorigenesis in recipient pseudo germ‐free mice. A) Experimental design for fecal microbiota transplantation for pseudo germ‐free mice (*n* = 15/each group). B) Observed sequence read counts, OTUs, and Shannon diversity of gut microbiota in pseudo germ‐free mice. C) Body weight of each group was recorded daily. D) LPS concentration in serum of M‐FMT‐AM (male mice received feces from male *Apc* ^Min/+^ mice), F‐FMT‐AM (female mice received feces from male *Apc* ^Min/+^ mice), M‐FMT‐AF (male mice received feces from female *Apc* ^Min/+^ mice), and F‐FMT‐AF (female mice received feces from female *Apc* ^Min/+^ mice) mice (*n* = 15/each group). E) Representative images of intercellular junctions of M‐FMT‐AM, F‐FMT‐AM, M‐FMT‐AF, and F‐FMT‐AF mice by transmission electron microscope. F) HE staining for pathologic diagnosis of mice colons. Quantitative analysis of pathologic score was calculated according to the following criteria: 0, normal; 1, LGD; 2, HGD; and 3, carcinoma. G) IHC staining of Ki‐67 and quantitative analysis of Ki‐67 index of colon section of pseudo germ‐free mice. H) The number of colon goblet cells was evaluated by PAS staining. The number of Paneth cells was evaluated by Lysozyme immunohistochemical staining. Six tissues were randomly selected from each section to calculate the percentage of positive cells per crypt. I,J) IHC for distribution of the adhesion molecule ZO‐1, Occludin and Claudin‐3 with quantitative analysis in colon tissues of M‐FMT‐AM, F‐FMT‐AM, M‐FMT‐AF, and F‐FMT‐AF mice. K) TNF‐*α* concentration in serum of pseudo germ‐free mice. L) IL‐10 concentration in serum of pseudo germ‐free mice. Data are expressed as mean ± SD. * *p* <  0.05, ** *p* < 0 .01, N.S. no significant.

To further validate the effect of sex‐biased gut microbes on sexual dimorphism in CRC tumorigenesis, we performed fecal microbiota transplantation (FMT) from male or female *Apc* ^Min/^ *^+^* mice (with high‐fat diet) into pseudo germ‐free mice treated with antibiotics (**Figure** **2**A). Mice randomly received feces from male *Apc* ^Min/^ *^+^* mice (FMT‐AM, including M‐FMT‐AM group for male recipients and F‐FMT‐AM group for female recipients) or female *Apc* ^Min/^ *^+^* mice (FMT‐AF, including M‐FMT‐AF group for male recipients and F‐FMT‐AF group for female recipients), respectively. Pseudo germ‐free mice were treated by antibiotics for 2 weeks. Five randomly selected fecal samples were subjected to 16S rRNA gene amplification sequencing. The results showed that sequence read counts, OTUs, and Shannon diversity of gut microbiota were significantly decreased in fecal samples of pseudo germ‐free mice (Figure 2B). FMT did not alter the body weight in pseudo germ‐free mice (Figure 2C). However, serum LPS concentration was higher in FMT‐AM mice than in FMT‐AF mice (Figure 2D). Meanwhile, there was no significant difference in LPS level between male and female pseudo germ‐free mice gavaged with same fecal samples (Figure 2D). The gut barrier structure under transmission electron microscopy showed the anabatic abnormalities of colonic intercellular junctions such as wider paracellular gap in in the FMT‐AM than in the FMT‐AF (Figure 2E). Consistently, more scattered small polyps and high‐grade dysplasia were observed in the FMT‐AM than in the FMT‐AF (Figure 2F). In addition, the increased Ki‐67‐positive cells (Figure 2G), decreased goblet cells (Figure 2H), and reduced tight junction protein expressions (ZO‐1, Occludin, and Claudin‐3) (Figure 2I,J) were observed in FMT‐AM mice, compared with FMT‐AF mice. Chronic inflammation is a recognized risk factor for CRC.^[^ ^17^ ^]^ ELISA results showed that the expression of pro‐inflammatory cytokine Tumor necrosis factor‐*α* (TNF‐*α*) (Figure 2K) was upregulated in FMT‐AM group, while anti‐inflammatory cytokine Interleukin‐10 (IL‐10) (Figure 2L) was upregulated in FMT‐AF group. Taken together, these results suggested that sex‐biased gut microbiota in CRC mice affected colorectal tumorigenesis by impairing gut barrier function.

### 2.4 Gut Microbiome is Sex‐Biased in CRC Mouse Model

**Figure 3:**
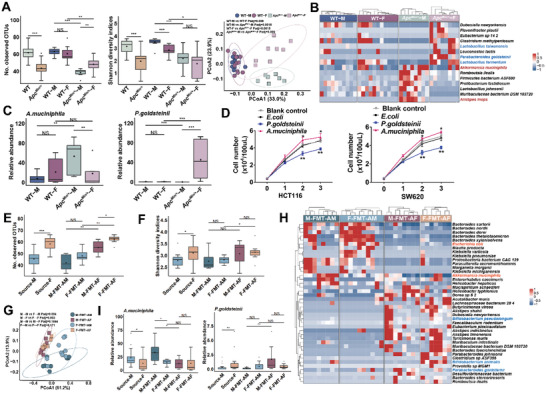
Gut microbiome is sex‐biased in CRC mouse model. A) Alpha‐diversity analysis of OUT and Shannon diversity, and PCoA analysis (beta diversity) of gut microbiota in WT‐M (*n* = 10), WT‐F (*n* = 11), *Apc* ^Min/+^‐M (*n* = 8), *Apc* ^Min/+^‐F mice (*n* = 9). B) Heatmap for gut microbiome of WT‐M, WT‐F, *Apc* ^Min/+^‐M, and *Apc* ^Min/+^‐F mice. The protective bacterial species are colored in blue and the potential pathogenic bacterial species are colored in red. C) The abundance of *Akkermansia muciniphila* and *Parabacteroides goldsteinii* in WT‐M, WT‐F, *Apc* ^Min/+^‐M, *Apc* ^Min/+^‐F mice. D) Growth curves of HCT116 and SW620 CRC cells coincubation with *P. goldsteinii*, *A. muciniphila*, *E. coli*, and blank control. E) Alpha‐diversity analysis of OUTs of gut microbiota among Source‐M (mice received feces from male *Apc* ^Min/+^), Source‐F (mice received feces from female *Apc* ^Min/+^), M‐FMT‐AM (*n* = 8), F‐FMT‐AM (*n* = 10), M‐FMT‐AF (*n* = 7), F‐FMT‐AF (*n* = 8). F) Alpha‐diversity analysis of Shannon diversity of gut microbiota Source‐M, Source‐F, M‐FMT‐AM, F‐FMT‐AM, M‐FMT‐AF, F‐FMT‐AF. G) PCoA analysis (beta diversity) of gut microbiota in M‐FMT‐AM, F‐FMT‐AM, M‐FMT‐AF, and F‐FMT‐AF mice. H) Heatmap for gut microbiome of M‐FMT‐AM, F‐FMT‐AM, M‐FMT‐AF, and F‐FMT‐AF mice. The protective bacterial species are colored in blue and the potential pathogenic bacterial species are colored in red. I) The abundance of *Akkermansia muciniphila* and *Parabacteroides goldsteinii* in Source‐M, Source‐F, M‐FMT‐AM, F‐FMT‐AM, M‐FMT‐AF, and F‐FMT‐AF mice. Data are expressed as mean ± SD. * *p* <  0.05, ** *p* < 0.01, *** *p* < 0.001, N.S. no significant. Dot plot reflects data points from independent experiment.

To identify the male‐biased pathogenic microbes and female‐biased probiotic microbes involved in CRC development, we performed shotgun metagenomic sequencing on faecal samples from male and female *Apc* ^Min/^ *^+^* mice and WT mice fed with high‐fat diet. Lower bacterial diversity and reduced bacterial richness were observed in *Apc* ^Min/^ *^+^* mice, compared with WT mice. Meanwhile, female *Apc* ^Min/^ *^+^* mice present increased bacterial diversity in compared with the male (**Figure** **3**A). Similarly, principal coordinates analysis (PCoA) results showed that the *β*‐diversity of microbes of WT‐M, WT‐F, *Apc* ^Min/^ *^+^*‐M, and *Ap*c^Min/^ *^+^*‐F exhibited a significant difference (Figure 3A). Different microbial composition was observed between male and female *Apc* ^Min/^ *^+^* with several differential bacterial taxa, and so was for WT mice. 29 and 21 sex‐biased bacterial species were identified in WT mice, and *Apc* ^Min/^ *^+^* mice (Table S2, Supporting Information). 15 bacteria species with specific differences between male and female *Apc* ^Min/^ *^+^* mice were screened (Figure 3B). The abundances of 2 potential pathogenic bacterial *species Alistipes inops* ^[^ ^18^ ^]^ and *Akkermansia muciniphila* ^[^ ^19^ ^]^ were significantly higher in male mice than female mice (Figure 3C and Figure S4, Supporting Information), whereas 3 probiotic bacterial species *Parabacteroides goldsteinii*,^[^ ^20^ ^]^ *Lactobacillus taiwanensis*,^[^ ^21^ ^]^ and *Lactobacillus fermentum* ^[^ ^22^ ^]^ were depleted in male mice. The differential abundance of *Akkermansia muciniphila* and *Parabacteroides goldsteinii* in the comparison of male versus female mice was investigated using quantitative polymerase chain reaction (qPCR) (Figure S5, Supporting Information). Additional coculture experiments demonstrated that *P. goldsteinii* inhibited cell growth, whereas *A. muciniphila* promoted cell growth in CRC cell lines (SW620 and HCT116) (Figure 3D), suggesting that the change of gut microbial composition in male *Apc* ^Min/^ *^+^* mice.

Meanwhile, we examined the composition of gut microbiome in recipient pseudo germ‐free mice. The shotgun metagenomic sequencing results showed that gut microbiome composition was significantly different between FMT‐AM mice and FMT‐AF mice. FMT‐AM mice exhibited lower bacterial diversity and richness than FMT‐AF mice (Figure 3E). Meanwhile, the number of OTUs in the feces of pseudo‐germ‐free mice receiving feces samples from female mice was significantly higher than that receiving feces samples from male mice (Figure 3F). PCoA analysis (beta diversity) showed significantly different clustering of gut microbiota among M‐FMT‐AM, F‐FMT‐AM, M‐FMT‐AF, and F‐FMT‐AF mice (Figure 3G). Different microbial composition was observed between FMT‐AM mice and FMT‐AF mice. Among these, *E. coli* and *A. muciniphila* were enriched while gut‐beneficial bacteria including *B. pseudolongum*, *B. animalis*, and *P. goldsteinii* were depleted in FMT‐AF mice (Figure 3H). The abundance of potential pathogenic bacterial species *A. muciniphila* was significantly higher in FMT‐AM mice than FMT‐AF mice, whereas the probiotic bacterial species *P. goldsteinii*, *was* depleted in FMT‐AM mice (Figure 3I). Consistent with this, the abundance of *A. muciniphila* also was significantly higher in *Apc* ^Min/^ *^+^*‐M mice, and *P. goldsteinii was* depleted in *Apc* ^Min/^ *^+^*‐M mice. Taken together, these results demonstrated increased the abundance of beneficial bacteria and decreased the abundance of harmful bacteria in female *Apc* ^Min/^ *^+^* mice.

### 2.5 Sex‐Biased Gut Metabolite LPC Enhances Cell Proliferation and Cell Junction Impairment

**Figure 4:**
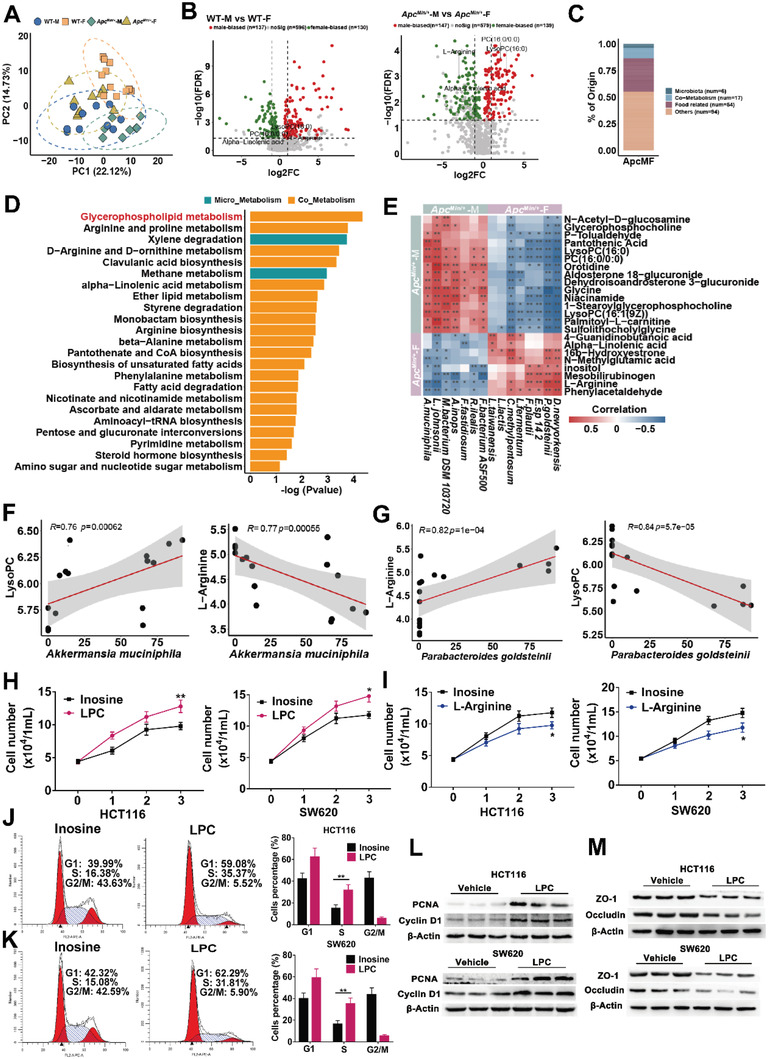
Sex‐biased gut metabolite LPC enhances cell proliferation and cell junction impairment. A) PCA plot for gut metabolomics analysis of WT‐M (*n* = 10), WT‐F (*n* = 11), *Apc* ^Min/+^‐M (*n* = 8), *Apc* ^Min/+^‐F mice (*n* = 9). B) Volcano plot for differential metabolites in comparison between WT‐M and WT‐F mice, or between *Apc* ^Min/+^‐M and *Apc* ^Min/+^‐F. C) The source of differential metabolites among *Apc* ^Min/+^‐M and *Apc* ^Min/+^‐F except for difference in WT. D) Pathway analysis of differentially enriched metabolites of microbial origin in *Apc* ^Min/+^‐M and *Apc* ^Min/+^‐F mice. E) Correlation analysis of the association of the sex‐biased microbes and metabolites. F) *A. muciniphila* was positively correlated with LPC, while *A. muciniphila* was negatively correlated with l‐Arginine. G) *P. goldsteinii* was positively correlated with LPC, while *P. goldsteinii* was positively correlated with l‐Arginine. H) Cell growth curves of CRC cell line HCT116 and SW620 treated with LPC and inosine (as negative control). I) Cell growth curves of CRC cell line HCT116 and SW620 treated with l‐Arginine and inosine. J,K) HCT 116 cells and SW620 cells treated with or without LPC were stained with propidium iodide (PI) and analyzed using flow cytometry. L) Expression levels of cell proliferation and cell cycle‐associated proteins PCNA and Cyclin D1, in HCT 116 and SW620 cells treated with LPC and inosine. M) Expression levels of gut barrier function‐associated proteins ZO‐1 and Occludin in HCT116 and SW620 cell lines. PCNA, proliferating cell nuclear antigen. Data are expressed as mean ± SD. * *p* < 0.05, ** *p *< 0.01. Dot plot reflects data points from independent experiment.

Gut microbiota could contribute to the development of CRC through the mediation of metabolites.^[^ ^23^ ^]^ To reveal metabolic changes induced by sex bias, we performed metabolic profiling of the feces samples from male and female *Apc* ^Min/^ *^+^* or WT mice fed with high‐fat diet. Principal component analysis (PCA) showed that gut metabolites were significantly different between male and female *Apc* ^Min/^ *^+^* or WT mice (**Figure** **4**A). We identified 267 sex‐biased metabolites in the WT mice, and 286 in *Apc* ^Min/^ *^+^* mice (Figure 4B). Among them, Phosphatidylcholine (PC) and LPC (the downstream metabolite of PC) were both the upregulated outlier metabolites in male mice (Figure 4B). l‐arginine and alpha‐linolenicacid were both the downregulated metabolites in male mice (Figure 4B). 23 sex‐biased metabolites of microbiological origin were identified in *Apc* ^Min/^ *^+^* mice but not in WT mice (Figure 4C and Table S3, Supporting Information). Among the pathways enriched by sex‐biased metabolites in *Apc* ^Min/^ *^+^*, glycerophospholipid metabolism was one top pathway with the greatest alteration in male mice (Figure 4D). PC and LPC were included in glycerophospholipid metabolism.

Integrative analysis was performed to determine the potential association between sex‐biased gut microbes and metabolites in male and female *Apc* ^Min/^ *^+^* mice (Figure 4E). We found that enrichment of *A. muciniphila* was positively correlated with LPC, while *A. muciniphila* was negatively correlated with l‐arginine (Figure 4F). Moreover, depletion of *P. goldsteinii* was positively correlated with LPC, while *P. goldsteinii* was positively correlated with l‐arginine (Figure 4G).

To explore the potential functional roles of sex‐biased metabolites in CRC development, 2 CRC cell lines (SW620 and HCT116) were treated with differential and nondifferential metabolites. Inosine, one of the unbiased metabolites between male mice and female mice, was used as negative control. Co‐culture experiments showed that LPC significantly promoted cell proliferation in CRC cell lines (Figure 4H), whereas l‐arginine significantly inhibited cell proliferation in 2 CRC cell lines (Figure 4I). Cell cycle analysis showed that LPC treatment accelerated cell cycle progression from G1 to S phase in HCT 116 and SW620 cells, compared with the control (Figure 4J,K). Consistent with these observations, upregulated protein expression of proliferating cell nuclear antigen (PCNA) and cyclin D1 were identified upon LPC treatment in CRC cell lines (Figure 4L). Furthermore, we examined whether LPC could affect epithelial barrier function. Significant decreased protein expressions of ZO‐1 and Occludin were observed in LPC‐treated HCT 116 and SW620 cells compared with their controls, inferring LPC could impair barrier function (Figure 4M). These results suggested that both the altered gut microbiota and their associated metabolites accelerated male‐associated colorectal tumorigenesis.

### 2.6 Sex‐Biased Gut Microbes in CRC Patients also Contributed to Sexual Dimorphism in Gut Barrier Function and Inflammation

We then compared the sex‐biased microbes between mouse and human, and among cohorts of CRC patients to identify common microbes potentially responsible for CRC tumorigenesis in human.

**Figure 5:**
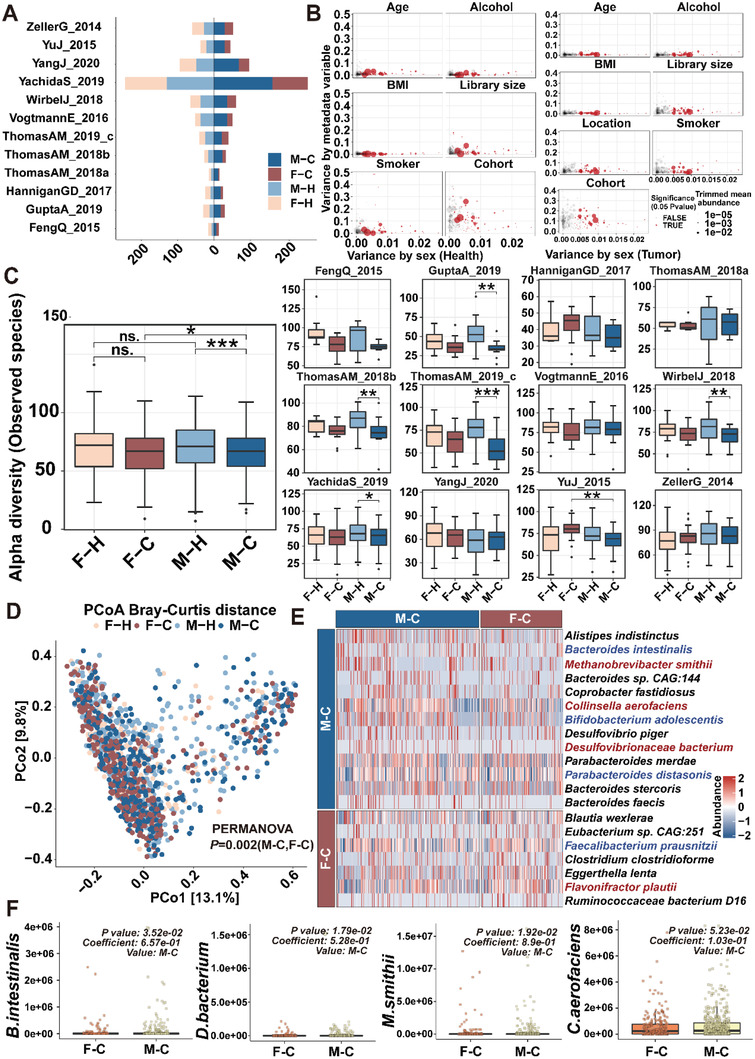
Gut microbiome is sex‐biased in CRC patients. A) The number of samples in the four groups from 12 cohorts with metagenomic data. B) Potential confounding of individual microbial species associations by patient demographics. Variance explained by sex was plotted against variance explained by different putative confounding factors (age, BMI, sex, cohort or other factors) for individual microbial species. Each species is represented by a dot proportional in size to its abundance; core microbial markers identified in the meta‐analysis are highlighted in red. C) Alpha diversity as measured with the number of observed species was computed in all cohorts. *P*‐values were computed using a two‐sided Wilcoxon test, while the overall *P* value was calculated using a two‐sided blocked Wilcoxon test in the left. D) Principal coordinate analysis (PCoA) of samples from all 12 cohorts based on Bray–Curtis distance, which shows that microbial composition was different between F‐H, F‐C, M‐H, and M‐C. E) Heatmap for gut microbiome of F‐C and M‐C. The protective bacterial species are colored in red and the potential pathogenic bacterial species are colored in blue. F) The abundance of *B. intestinalis*, *D. bacterium*, *M. smithii*, and *C. aerofaciens* in F‐C and M‐C. The significance threshold is the default *P*‐value threshold in MaAsLin2. M‐C: male CRC patients; F‐C: female CRC patients; M‐H: male healthy individuals; F‐H: female healthy individuals. Dot plot reflects data points from independent experiment.

To investigate whether there is also a sex bias in the gut microbiome of colorectal cancer (CRC) patients, we collected 12 shotgun metagenomic sequencing datasets from healthy individuals and CRC patients and conducted a meta‐analysis (**Figure** **5**A). As these datasets were different in biology and technology, we quantified the impact of cohort‐associated heterogeneity on microbiota composition. We compared the “cohort” factor with other potential confounders (age, BMI, and location, etc.) and found that the “cohort” factor had a major effect on comparing sex‐biased microbiota composition (Figure 5B), mainly due to differences in populations and DNA extraction protocols.^[^ ^24^, ^25^, ^26^ ^]^ To evaluate the differences in the microbiota of male and female CRC patients, we performed a meta‐analysis by integrating 12 shotgun metagenomic sequencing datasets and assessed alpha and beta diversity, treating the “cohort” factor as a confounding factor. We observed a significant difference in alpha diversity between the M‐C and F‐C groups after integrating the data (Figure 5C). Microbiota composition differences were observed among healthy men, healthy women, male CRC patients, and female CRC patients using PcoA (Figure 5D). We mainly examined sex‐biased gut microbiota in CRC patients. A total of 20 gut microbes with differential abundance were identified in the comparison of male versus female CRC patients (Figure 5E). The abundance of 4 potential pathogenic bacterial species including *Collinsella aerofaciens* and *Desulfovibrionaceae bacterium* were significantly higher in male CRC patients than in female counterparts (Figure 5F).

**Figure 6:**
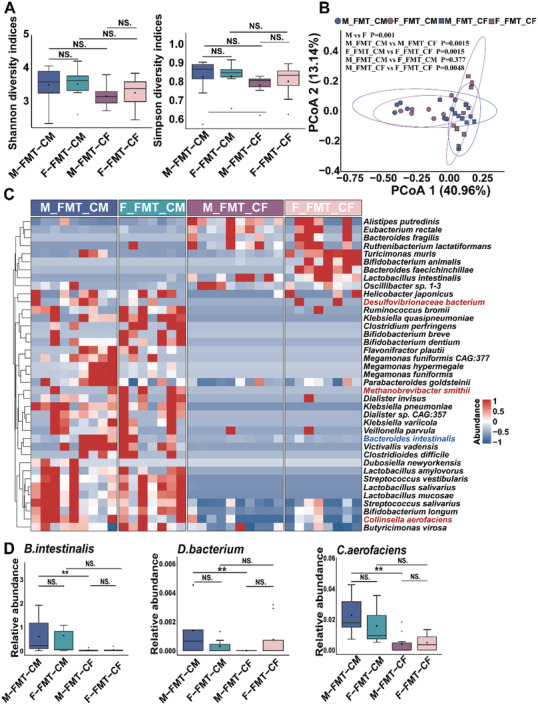
Gut microbiome is sex‐biased in recipient pseudo germ‐free mice gavaged with fecal samples from CRC patients. A) Alpha‐diversity analysis of Shannon diversity and Simpson diversity, and B) PCoA plot (beta diversity) of gut microbiota in M‐FMT‐CM (*n* = 9; male mice received feces from male patients), F‐FMT‐CM (*n* = 7; female mice received feces from male patients), M‐FMT‐CF (*n* = 9; male mice received feces from female patients), and F‐FMT‐CF (*n* = 8; female mice received feces from female patients) mice. C) Heatmap for gut microbiome of M‐FMT‐CM, F‐FMT‐CM, M‐FMT‐CF, and F‐FMT‐CF mice. The protective bacterial species are colored in red and the potential pathogenic bacterial species are colored in blue. D) The abundance of *B. intestinalis*, *D. bacterium*, *M. smithii*, and *C. aerofaciens* in M‐FMT‐CM, F‐FMT‐CM, M‐FMT‐CF, and F‐FMT‐CF mice. Data are expressed as mean ± SD. * *p* < 0.05, ** *p* < 0.01, N.S. no significant. Dot plot reflects data points from independent experiment.

To further investigate the effects of human sex‐biased gut microbial composition difference on CRC tumorigenesis, we gavaged male or female CRC patient feces to pseudo germ‐free mice (Figure S6A, Supporting Information). Fecal microbiota transplantation (FMT) did not alter the body weight in pseudo germ‐free mice (Figure S6B, Supporting Information). However, serum LPS concentration was higher in mice receiving CRC male patient feces (FMT‐CM, including M‐FMT‐CM for male recipients and F‐FMT‐CM for female recipients) mice than in mice receiving CRC female patient feces (FMT‐CF, including F‐FMT‐CM for male recipients and F‐FMT‐CF for female recipients) mice (Figure S6C, Supporting Information). Meanwhile, no significant difference in LPS level was observed between male and female pseudo germ‐free mice gavaged with same fecal samples (Figure S6C, Supporting Information). Consistently, more scattered small polyps and high‐grade dysplasia were observed in the FMT‐CM than in the FMT‐CF (Figure 6D, Supporting Information). In addition, the increased Ki‐67‐positive cells (Figure S6E, Supporting Information), decreased goblet cells (Figure S6F, Supporting Information), widened paracellular gap (Figure S6G, Supporting Information), and reduced tight junction protein expressions (ZO‐1, Occludin, and Claudin‐3) were observed in FMT‐CM mice, compared with FMT‐CF mice (Figure S6H, Supporting Information). ELISA results showed that the expression of anti‐inflammatory cytokine IL‐10 was up‐regulated in FMT‐CF group (Figure S6I, Supporting Information), while the expression of pro‐inflammatory cytokine TNF‐*α* was up‐regulated in FMT‐CM group (Figure S6J, Supporting Information). Overall, these results suggested that gut microbiota in male CRC patients, as that in male *Apc* ^Min/^ *^+^* mice, accelerated colorectal tumorigenesis by impairing gut barrier function and increasing inflammation, which was in line with the results of mouse FMT experiments.

We further investigated the composition of gut microbiota in recipient pseudo germ‐free mice through shotgun metagenomic sequencing on feces samples and found the significant difference in gut microbiota composition between FMT‐CM mice and FMT‐CF mice. No significant differences in bacterial diversity and richness were found between FMT‐CM mice than FMT‐CF mice (**Figure** **6**A). However, PCoA showed that the *β*‐diversity of feces samples from M‐FMT‐CM, F‐FMT‐CM, M‐FMT‐CF, and F‐FMT‐CF had a significant difference (Figure 6B). Different microbial composition was observed between FMT‐CM and FMT‐CF with several differential bacterial species (Figure 6C). Among them, the abundances of potential pathogenic bacterial species *D. bacterium* and *C. aerofaciens* were significantly higher in M‐FMT‐CM mice than M‐FMT‐CF mice (Figure 6D).

All the results above indicated that the intestinal barrier function impairment in pseudo germ‐free mice gavaged with feces from male patients was more severe than that from female patients, which might be partially attributed to the aggravated gut microbial dysbiosis. Therefore, it could be concluded that sex‐biased gut microbes, although species‐ and cohort‐dependent, were involved in sexual dimorphisms in CRC tumorigenesis.

### 2.7 Male‐Biased Gut Metabolites Aggravated Colorectal Tumorigenesis through the Glycerophospholipid Metabolism Pathway in Both Human and Mouse

The common contribution of distinct mouse and human sex‐biased gut microbiomes to the sex dimorphism in CRC could be explained by the same function sex‐biased gut metabolites generated from distinct sex‐biased microbes. To test this speculation, we compared the sex‐biased gut metabolites of pseudo germ‐free mice receiving feces from human and mouse CRC models.

**Figure 7:**
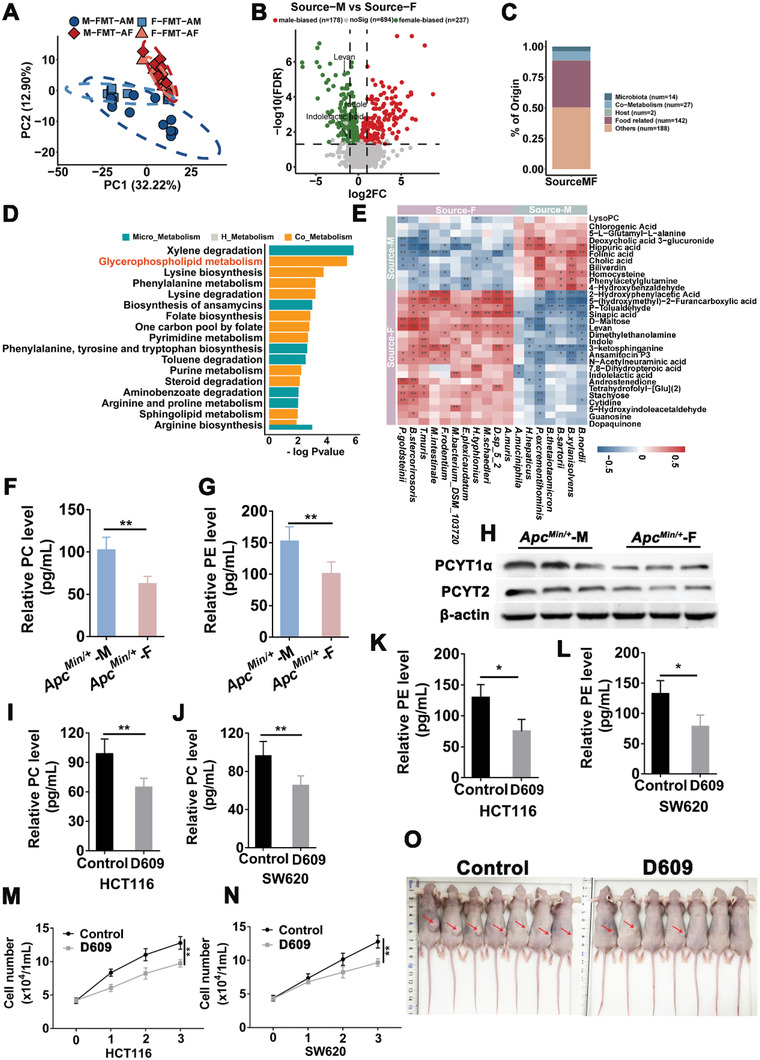
Male‐biased gut metabolites aggravated colorectal tumorigenesis through the glycerophospholipid metabolism pathway. A) PCA plot for gut metabolomics analysis of M‐FMT‐AM, F‐FMT‐AM, M‐FMT‐AF, and F‐FMT‐AF mice (*n* = 10/each group). B) Volcano plot for differential metabolites in comparison between source‐M (mice received feces from male *Apc* ^Min/+^ mice) and source‐F (mice received feces from female *Apc* ^Min/+^ mice) mice and the metabolites were screened for differences in origin that were stable in the two sexes as candidates. C) The source of differential metabolites among selected candidates. D) Pathway analysis of differentially enriched metabolites of microbial origin in source‐M and source‐F mice. E) Correlation analysis of the association of the sex‐biased microbes and metabolites in source‐M and source‐F mice. F,G) Expression level of PC and PE in colon tissues of *Apc* ^Min/^ *^+^*‐M and *Apc* ^Min/^ *^+^*‐F mice using ELISA. H) Expression protein level of PCYT2 and PCYT1*α* in colon tissues of *Apc* ^Min/^ *^+^*‐M and *Apc* ^Min/^ *^+^*‐F mice. I–L) Expression level of PC and PE in CRC cell lines treated with Vehicle (control) and D609. M,N) Cell growth curves of CRC cell line HCT116 and SW620 treated with Vehicle and D609. O) Representative images for tumor formation in nude mice (*n* = 7/each group) treated with Vehicle and D609. Data are expressed as mean ± SD. * *p* < 0.05, ** *p* < 0.01. Dot plot reflects data points from independent experiment.

We performed metabolic profiling of gut metabolites of feces samples from pseudo germ‐free mice gavaged with feces from male or female *Apc* ^Min/^ *^+^* mice. Orthogonal partial least squares discriminant analysis showed that fecal metabolic profiles of FMT‐AM mice was significantly different from those of FMT‐AF mice (**Figure** **7**A). The differential metabolites were identified in the comparison of FMT‐AM mice versus the FMT‐AF mice (Figure 7B). Among them, 43 were derived from gut microbes (Figure 7C and Table S4, Supporting Information). The sex‐biased metabolites were enriched or depleted in different metabolomic signaling pathways. Among these pathways, glycerophospholipid metabolism was one of the top pathways enriched in FMT‐AM mice, similar as that in male *Apc* ^Min/^ *^+^* mice (Figure 7D). Glycerophospholipids have been reported to be biomarkers for monitoring CRC patients.^[^ ^27^, ^28^ ^]^ To reveal potential relationship between microbiota and metabolites, we performed correlation analysis between bacteria and metabolites by partial Spearman correlation, and found that the potential pathogenic bacterial *A. muciniphila* in FMT‐AM was positively correlated with LPC (Figure 7E).

To reveal sex‐induced metabolic changes in CRC patients, we performed metabolic profiling of the feces samples from F‐FMT‐CM, M‐FMT‐CM, M‐FMT‐CF, and F‐FMT‐CF mice. The unsupervised PCA results showed that gut metabolites differed significantly among pseudo germ‐free mice gavaged with different feces from male or female patients (Figure S7A, Supporting Information). The differential metabolites were identified in the comparison of FMT‐CM mice versus FMT‐CF mice (Figure S7B, Supporting Information). We also identified the source of these differential metabolites. Among these differential metabolites, 19 were derived from gut microbes (Figure S7C and Table S5, Supporting Information). The differential metabolites between FMT‐CM mice and FMT‐CF mice were enriched in different metabolomic signaling pathways. Among these pathways, glycerophospholipid metabolism was also one of the pathways enriched in FMT‐CM (Figure S7D, Supporting Information), which was consistent with the enriched pathway found in the FMT‐AM mice metabolome. Furthermore, we performed integrative analysis to examine the potential relationship between altered gut microbes and metabolites (Figure S7E, Supporting Information).

Glycerophospholipid metabolism pathway is the only conserved metabolic pathway in which male‐biased metabolites are enriched in human and mouse. Activation of glycerophospholipid metabolism pathway accelerates the progression of colorectal cancer.^[^ ^29^, ^30^, ^31^ ^]^ Phosphatidylcholine (PC) and phosphatidylethanolamine (PE) were abnormally accumulated in patients with colorectal cancer.^[^ ^30^ ^]^ To investigate the role of GPL in CRC development, we measured the levels of PC and PE in the colon tissues of *Apc* ^Min/^ *^+^* mice. Our results revealed that PC and PE levels in the colon tissues of male *Apc* ^Min/^ *^+^* mice were significantly higher than those in female mice (Figure 7F,G). Furthermore, the protein expression of PCTYT1*α* and PCYT2, critical rate‐limiting enzymes in GPL metabolism, were significantly elevated in male *Apc* ^Min/^ *^+^* mice (Figure 7H). GPL metabolism plays an important role in the proliferation and tumor progression of colorectal cancer cells. Blocking GPL metabolism in cancer cells has become a potential treatment for colorectal cancer.^[^ ^32^, ^33^, ^34^, ^35^ ^]^ In this study, GLP metabolic pathway inhibitor D609 was selected for its effect on the proliferation of two colorectal cancer cell lines (HCT116 and SW620), and the results showed that D609 significantly the reduced the level of PC and PE (Figure 7I–L), inhibited the proliferation of CRC cell lines (Figure 7M,N). Moreover, in the tumor formation experiment of nude mice, we found that D609 significantly inhibited the formation of subcutaneous tumors in nude mice (Figure 7O). These results suggested that gut metabolites were sex‐biased in both human and mouse CRC models, and that the male‐biased gut metabolites aggravated colorectal tumorigenesis through the glycerophospholipid metabolism pathway in both human and mice.

## 3 Discussion

This study revealed sexual dimorphism in colorectal tumorigenesis in different mouse models. Our results in mouse model are supported by the previous worldwide reports on sexual dimorphism in CRC that men display higher incidence and mortality rates of CRC than women.^[^ ^5^, ^36^, ^37^ ^]^ Our data showed there was a significant difference in the number and size of tumors between male *Apc* ^Min/^ *^+^* (standard diet and high‐fat diet) mice or male AOM/DSS mice and female counterparts, but this difference disappeared after antibiotic‐induced gut microbiome depletion. The greatest phenotype differences during CRC progression were observed between male and female *Apc* ^Min/^ *^+^* mice fed with high‐fat diet, and thus we selected this model for subsequent analysis.

It has been reported that intestinal barrier dysfunction contributes to CRC development.^[^ ^38^ ^]^ The damage to mucosal barrier can result in unlimited entry of commensal microbes including pathogens to the lamina propria, or even to the bloodstream, thus accelerating CRC development.^[^ ^39^ ^]^ Further, we explored the possible difference in epithelial barrier function between male and female *Apc* ^Min/^ *^+^* mice. LPS, an abundant component in gut microbiome, is involved in CRC progression and metastasis.^[^ ^40^ ^]^ Our ELISA results showed the increased serum LPS level in male mice, which was consistent with the report on the increased LPS level in patients with CRC and colonic hyperpermeability.^[^ ^41^ ^]^ Moreover, our results displayed wider paracellular gap and lower protein expression of ZO‐1, Occludin, and Claudin‐3 (gut barrier function‐related markers) in male mice than female mice, suggesting more severe impairment of gut barrier function in male mice relative to female mice.

Increasing evidence has revealed that gut microbiome plays a critical role in CRC tumourigenesis.^[^ ^42^, ^43^, ^44^ ^]^ Some previous studies showed that transplantation of feces from CRC patients can promote tumourigenesis in germ‐free mice and AOM‐treated mice,^[^ ^42^, ^45^ ^]^ and that transplantation of feces from AOM/DSS mice to germ‐free mice led to accelerated tumor development, compared with that from healthy mice.^[^ ^46^ ^]^ Our results further demonstrated that pseudo germ‐free mice gavaged with fecal samples from male or female mice and CRC patients on colonic mucosa in pseudo germ‐free mice. We found that the alterations in intestinal barrier function depended on the sex of fecal donators (mice or patients), rather than the sex of pseudo germ‐free mice themselves. Pseudo germ‐free mice gavaged with feces from male mice or male CRC patients exhibited significantly enhanced cell proliferation, gut barrier dysfunction, and intestinal inflammation. These findings jointly suggested that gut microbiome may play an essential role in colorectal tumorigenesis.

Multiple evidence show that sexual dimorphism in CRC is modulated by the tumor microenvironment,^[^ ^47^ ^]^ estrogen,^[^ ^48^, ^49^ ^]^ and X‐linked genes.^[^ ^50^ ^]^ Considering that CRC patients presented intestinal microbial disorder relative to healthy humans, and that sex is a major contributor to the differences in gut microbiome,^[^ ^45^, ^51^ ^]^ this study analyzed the sex‐biased colorectal tumorigenesis from the perspective of gut microbiome. Our data showed that male and female *Apc* ^Min/^ *^+^* mice exhibited distinct microbiota composition. *A. inops* and *A. muciniphila* were significantly enriched in male *Apc* ^Min/^ *^+^* mice and pseudo germ‐free mice gavaged with fecal samples from male *Apc* ^Min/^ *^+^* mice. *A. mucinipila* is an important gram‐negative anaerobic bacterium, and it can degrade mucin in gut.^[^ ^52^ ^]^ This bacterium is positively correlated with colonic tumor burden.^[^ ^53^ ^]^ Our results showed that *A. mucinipila* was enriched both in male *Apc* ^Min/^ *^+^* mice and pseudo germ‐free mice receiving male *Apc* ^Min/^ *^+^* feces. In addition, our co‐culture experiments showed that *A. mucinipila* promoted CRC cell growth, which was in line with the previous report that *A. mucinipila* promoted the formation of CRC in mice by increasing the early‐stage inflammation and the intestinal epithelial cell proliferation.^[^ ^19^ ^]^ Overall, these findings collectively suggest that *A. mucinipila* acts as a potential pathogen in colorectal tumorigenesis. In this study, 3 probiotic bacteria *P. goldsteinii*, *L. taiwanensis*, and *L. fermentum* were depleted in male mice and pseudo germ‐free mice gavaged with fecal samples from male *Apc* ^Min/^ *^+^* mice, of which *P. goldsteinii* was found to inhibit CRC cell growth in vitro. *P. goldsteinii* has been reported to generate anti‐inflammatory lipopolysaccharide, thus significantly ameliorating intestinal inflammation and enhancing cellular mitochondrial and ribosomal activities in colon.^[^ ^54^ ^]^ Additionally, *P. goldsteinii* is also involved in maintaining intestinal epithelial barrier function to attenuate CRC development.^[^ ^55^ ^]^ These findings collectively indicate that the depletion of probiotic bacteria and enrichment of oncogenic bacteria in male gut microbiome contribute to male‐biased CRC development.

In addition to gut microbiota, small‐molecule metabolites produced by dietary food and commensal bacteria could also contribute to colorectal tumorigenesis.^[^ ^23^ ^]^ Gut microbiota‐associated metabolites could both agonise and antagonise their cognate receptors to reduce or exacerbate intestinal tumor development.^[^ ^6^ ^]^ We unveiled the role of sex‐biased gut metabolites in colorectal tumorigenesis. There existed an obvious difference in metabolomic profile between male and female mice. We found that l‐arginine, an essential amino acid, was depleted in male mice, and that l‐arginine could significantly inhibit CRC cell growth, which was in line with the previous reports that l‐arginine are necessary for cell growth and differentiation‚ and it can reduce crypt cell hyper proliferation in CRC patients by increasing the NO concentration and decreasing ornithine decarboxylase (ODC) activity.^[^ ^56^, ^57^, ^58^ ^]^ More importantly, we found that the elevated level of LPC was positively correlated with *A. muciniphila* in male mice, both of which promoted CRC development in male mice. LPC is the downstream metabolite of PC, and the pro‐tumorigenic function of PC has been reported.^[^ ^59^ ^]^ LPA and LPC are both bioactive lipolytic products of phospholipase A2 group 1B (PLA2g1b) which could be modulated by gut microbiota.^[^ ^60^, ^61^ ^]^ In addition, increasing evidence supports that LPC can promote colorectal tumorigenesis,^[^ ^62^, ^63^ ^]^ which is in accordance with our results that LPC promoted the proliferation of CRC cells and accelerated their cell cycle. Overall, our findings suggest that male‐biased gut microbiota dysregulates gut metabolism, thus elevating oncogenic LPC levels, promoting cell proliferation, and impairing gut barrier function, eventually resulting in the acceleration of CRC development.

In conclusion, our study uncovers sexual dimorphism in colorectal tumorigenesis in multiple mouse models. Male and female mice exhibited distinct gut microbiota composition. Male mice displayed significant enrichment of harmful bacteria and depletion of probiotic bacteria, thus deteriorating gut barrier function, ultimately accelerating their CRC tumorigenesis and increasing their mortality. Therefore, modulating sex‐biased gut microbiome and metabolites could be a precise sex‐targeting therapeutic strategy for the prevention and treatment of CRC.

## 4 Experimental Section

### Participants

Eligible patients diagnosed as CRC by colonoscopy at the Endoscopy Centre at the Department of Gastroenterology and Hepatology, Hubei Cancer Hospital, China were enrolled in the study. Patient exclusion criteria were referenced in published articles.^[^ ^42^, ^45^, ^64^ ^]^ All Subjects information was provided in Table S1, Supporting Information. All donors had undergone rigorous screening and underwent informed consent for stool donation. The human study conformed “International ethical guidelines for biomedical research involving human subjects (2002)” developed by Council For International Organizations Of Medical Sciences (CIOMS) in collaboration with World Health Organization (WHO), which was approved by the ethics committee of Hubei Cancer Hospital and Huazhong Agricultural University.

### Conventional CRC Mouse Models

All mice used in this paper were in a predominant C57Bl/6L background (*n* = 320, every cage with 3–4 mice per cage, the mice at a similar weight were randomly assigned to each group). The mice were housed in a temperature controlled, specific pathogen free environment, with a 12 h light/dark cycle. Male and female C57BL/6L mice at 8 weeks old were intraperitoneally injected with 10 mg kg^−1^ AOM (Merck, Darmstadt, Germany), followed by three cycles of DSS (MP Biomedicals, Solon, OH) administration to mimic colitis‐associated CRC (*n* = 40, half male and half female). For each cycle, mice were allowed free access to drinking water supplemented with 2.0% DSS for 7 days, followed by 14 days of regular water. Male and female *Apc* ^Min/+^ mice, which can faithfully recapitulate the human familial adenomatous polyposis, was used as a mouse model of spontaneous CRC.^[^ ^65^ ^]^ The *Apc* ^Min/+^ mice were divided into three groups (half male and half female): high‐fat diet group (*n* = 40), standard diet group (*n* = 40), and antibiotics cocktail group (*n* = 20). Male and female C57Bl/6L as control group (*n* = 60, half male and half female). Drinking water was supplemented with antibiotics cocktail (0.2 g L^−1^ of ampicillin, neomycin, and metronidazole and 0.1 g L^−1^ of vancomycin) for 2 weeks, every other 2 weeks, until the end of experiment, to deplete the gut microbiota. Mice were harvested at day 126 or day 176 for AOM/DSS or *Apc* ^Min/+^ models, respectively. All procedures adhered to the guidelines approved by the Animal Experimentation Ethics Committee of the Huazhong Agricultural University.

### Pseudo Germ‐Free Mice Models

Pseudo germ‐free mice models (*n* = 120; half male and half female) were established by anhydrous antibiotics cocktail (0.2 g L^−1^ of ampicillin, neomycin, and metronidazole and 0.1 g L^−1^ of vancomycin) gavage for a period of 2 weeks.^[^ ^66^ ^]^ In addition, antibiotics cocktail was fed in water to the mice for a period of 2 weeks. Fecal bacteria were tested by 16S ribosomal RNA gene sequencing to verify the successful establishment of pseudo‐germ‐free mice. All pseudo‐germ‐free mice were randomly divided into four groups with 30 mice each (half male and half female). Different groups were gavaged fecal samples from male *Apc* ^Min/^ *^+^* mice (FMT‐AM), female *Apc* ^Min/^ *^+^* mice (FMT‐AF), male patients with CRC (FMT‐CM), and female patients with CRC (FMT‐CF). The animal use protocol had been reviewed and approved by the Animal Ethical and Welfare Committee (AEWC) of Huazhong Agricultural University.

### Nude Mice

6‐week‐old specific pathogen‐free female BALB/c nude mice were provided by a national rodent seed center (Hunan SJA Laboratory Animal Co., Ltd.) and maintained in an isolated clean room held at a regulated temperature (25 ± 2 °C) and humidity (≈40–50%). The mice were housed under a 12 h/12 h light/dark cycle and fed ad libitum with rodent diet and water. All protocols were approved and performed according to the guidelines of Huazhong Agricultural University.

### Establishment of a Subcutaneously Implanted Tumor Model

Inoculation method for the HCT 116 cell suspensions. The cells were collected in the exponential phase and digested into single‐cell suspensions. Then, the concentration of these single‐HCT116 cell suspensions was adjusted to 3 × 10^6^ cells mL^−1^. Anesthetized nude mice were disinfected with 75% alcohol and then inoculated with 200 µL cell suspensions in the middle of the right armpit. The mice were disinfected again and placed in laminar air flow rack while their physical signs were monitored. Nude mice were given either normal saline (control) or a glycerophospholipid metabolic pathway inhibitor (D609, HY‐70072, MCE) by gavage 2 h before injection and the same treatment at the same time for the following 16 days.

### Bacteria and CRC Cell Coculture

CRC cells were seeded at a 96‐well plate (5000 cells per well) in Dulbecco's modified Eagle medium (Gibco BRL, Grand Island, NY) supplemented with 10% fetal bovine serum. Cells were exposed to *Akkermansia muciniphila* or *Parabacteroides goldsteinii* with a multiplicity of infection of 100 for 4 h under anaerobic conditions. The medium containing the bacteria was then replaced with Dulbecco's modified Eagle medium supplemented with 10% fetal bovine serum, 1% penicillin–streptomycin, and 40 µg mL^−1^ gentamycin. The coculture was performed for up to 3 days. Cells were trypsinized and the number of cells was counted every day.

### Determination of Tumor Number and Volume from Mouse Models

The whole intestine was isolated and rinsed with ice‐cold sterile PBS solution. Solid neoplastic lesions were counted for tumor number and measured for tumor volume (major diameter × minor diameter^2^/2). The proximal tissue of each intestinal segment was snap frozen in liquid nitrogen and kept at −80 °C later and the distal tissue was fixed with 4% paraformaldehyde. Sections (4 mm) were stained with hematoxylin and eosin for histologic examination. Pathological types of specimens were diagnosed by two experienced pathologists who were unaware of the treatment allocation of the mice.

### Immunohistochemistry Staining

Paraffin‐embedded intestinal tissue cut into 4 µm slices by a microtome were also subjected to immunostaining for detecting the expressions of Ki‐67, Claudin‐3, and Occludin with primary antibodies of Ki‐67 (catalog number 16 667; Abcam), ZO‐1 (catalog number 33‐9100; Thermo Fisher Scientific), Claudin‐3 (catalog number 34‐1700; Thermo Fisher Scientific), and Occludin (catalog number PA5‐30230, Thermo Fisher Scientific), and six areas randomly selected from each section were viewed at the tumor tissue. The percentage of positive cells in each field was calculated by Image J.

### PAS Staining

Colon sections were incubated with 1% periodic acid solution (Sigma‐Aldrich) for 10 min, and with Schiff reagent (Sigma‐Aldrich) for 40 min subsequently, and followed by haematoxylin dye for 5 min.

### Serum LPS, TNF‐*α*, and IL‐10 Quantification

The serum LPS, TNF‐*α*, and IL‐10 level were measured with an ELISA kit (LPS: catalog number RK04263, ABclonal; TNF‐*α*: catalog number RK00027, ABclonal; IL‐10: catalog number RK00016, ABclonal). All testing procedures were performed according to the manufacturer's instructions.

### Tissues or Cells PC and PE Level Quantification

The serum PC and PE level were measured with an ELISA kit (PC: catalog number A12218, Thermo Fisher Scientific; PE: catalog number ZC‐38479, ZciBio). All testing procedures were performed according to the manufacturer's instructions.

### RNA Extraction and Realtime‐PCR

Total RNA was extracted using the RNeasy mini kit, and cDNA reverse transcription was carried out using the TIAN Script RT Kit according to the manufacturer's instructions. The oligonucleotide primers for target genes (ZO‐1Forward primer: 5′‐GGGCCATCTCAACTCCTGTA‐3′, Reverse primer: 5′‐AGAAGGGCTGACGGGTAAAT‐3′; Claudin‐3 Forward primer: 5′‐CCTGTGGATGAACTGCGTG‐3′, Reverse primer: 5′‐GTAGTCCTTGCGGTCGTAG‐3′; Occludin Forward: primer:5′‐ACTATGCGGAAAGAGTTGACAG‐3′, Reverse primer: 5′‐GTCATCCACACTCAAGGTCAG‐3′). The relative mRNA expression was performed using a standard ∆∆*CT* method to calculate fold‐changes normalized to housekeeping genes for each sample.

### Western Blotting

Intestinal tumor tissue was adequately homogenized on ice in a mixture of RIPA, PMSF, and protease inhibitors. Centrifugation was spun at 13 000 rpm to collect the supernatant containing total protein. Protein concentration was measured using detergent compatible protein assay (BIO‐RAD, Hercules, CA). Then, 40 mg of protein was separated by 5% upper gel and 12% lower gel and transferred onto polyvinylidene difluoride membranes (GE Healthcare, Piscataway, NJ). The primary anti‐*β*‐catenin (ab32572, Abcam,1:5000), ZO‐1 (33‐9100; Thermo Fisher Scientific, 1:1000), Claudin‐3 (34‐1700; Thermo Fisher Scientific, 1:1000), Occludin (PA5‐30230; Thermo Fisher Scientific, 1:1000), PCNA (13 110; Cell Signaling, 1:2000), Cyclin D1 (2922; Cell Signaling, 1:1000), PCYT2 (A15309, ABclonal, 1:1000) and PCYT1*α* (ab109263, Abcam, 1:1000) were applied. Secondary antibodies were horseradish peroxidase conjugated anti‐rabbit or anti‐mouse. Chemiluminescence signals were detected by the ECL detection kit. The intensity of Western blotting images was determined by Image J.

### Transmission Electron Microscopy

Small pieces of colon tissues were collected and fixed in 2.0% glutaraldehyde in 0.1 mol L^−1^ sodium cacodylate (Electron Microscopy Sciences, Hatfield, PA). Ultrathin sections were prepared on a Reichert Ultracut E ultramicrotome. The ultrastructure of the tissues was examined using a Philips CM100 transmission electron microscope.

### rDNA Amplicon Sequencing

Fresh feces of pseudo germ‐free mice were collected for 16S rDNA Amplicon sequencing which was performed by the Shanghai Majorbio Bio‐pharm Technology (Shanghai, China). The DNA of fecal bacteria was extracted by using a DNA extraction kit. Its integrity and size were confirmed by 1% agarose gel electrophoresis. The hypervariable region V3–V4 of the bacterial 16S rRNA gene were amplified with primer pairs 338F (5′‐ACTCCTACGGGAGGCAGCAG‐3′) and 806R (5′‐GGACTACHVGGGTWTCTAAT‐3′) by an ABI GeneAmp 9700 PCR thermocycler (ABI, CA, USA). The operational taxonomic units (OTUs) were assigned for the 16S rRNA gene sequences with a threshold of 97% for paired recognition, and were classified using gg‐13‐8‐99‐nb‐classifier in Qiime2.^[^ ^4^ ^]^ The *α* diversity estimates were calculated by the Shannon and OTUs number index.

### Shotgun Metagenome Sequencing and Taxonomic Annotation

Mice fecal DNA was extracted using the DNeasy PowerSoil Kit (QIAGEN, Valencia, CA). Shotgun metagenomic sequencing of mice fecal DNA was performed on Illumina HiSeq 2000 platform (Illumina, Shenzhen, BGI). The sequences from the obtained fecal metagenomic shotgun sequencing, were subjected to quality filtering using “trimmomatic‐options” in Kneaddata (v0.10.0), and reads less than 50 nucleotides were discarded. The filtered reads were then aligned with the mouse genome (C57BL), and the mouse DNA was removed using bowtie2. MetaPhlAn3 (v3.0.14) was used to quantify the taxonomic composition of microbial communities across all metagenomic samples, while HUMANn3 (v3.0.1) was used to analyze pathway and gene family abundance.^[^ ^67^ ^]^ OTUs and Shannon index were used to assess alpha diversity and the unweighted unifrac distance was used to calculate the difference in beta diversity between samples. Mann–Whitney U test was used to screen for differential bacteria, including those that met the criteria (*P* < 0.05) as candidates. The public OTUs profiles generated for the 11 public cohorts,^[^ ^68^, ^69^, ^70^, ^71^, ^72^, ^73^, ^74^, ^75^, ^76^ ^]^ along with their metadata and the two newly sequenced cohorts, are available through the curated Metagenomic Data R package.^[^ ^77^ ^]^ Besides, the YangJ_2020 dataset used Kneaddata for quality filtering, MetaPhlAn3 (v3.0.14) for quantitative profiling and HUMANn3 (v3.0.1) for analyzing pathway and gene family abundance.^[^ ^78^ ^]^ Samples that were adenoma and samples with other diseases, were discarded. Samples with low alignment reads (≤1 000 000) were then excluded. Outliers and suspected contaminated cases were also removed, including samples with high species content (species read counts ≥50% of the total) and low species content (species read counts ≤10% of the total × 1/*n*; *n* is the number of samples with different disease states in each cohort). Moreover, Species with low abundance were discarded (species read counts ≤0.001 of the total). Finally, 1424 samples and 344 species were retained, including 707 healthy samples (379 males and 328 females) and 717 CRC patients (457 males and 260 females).

### Metabolomics Profiling for Fecal Samples

A total of 50 mg of each fecal sample was weighed for the metabolomics study. The metabolites were extracted using 80% cold methanol. After centrifugation at 21 500 g for 15 min at 4 °C, the supernatant was collected for the high‐performance liquid chromatography‐mass spectrometry analysis. The instrumental analysis was performed on an Agilent 1290 Infinity UPLC system (Agilent Technologies, Santa Clara, CA) coupled to a Sciex TripleTOF 6600 mass spectrometer (Q‐TOF, AB Sciex, Toronto, Canada). The chromatographic separation was achieved on a Waters BEH Amide column (2.1 mm × 100 mm; 1.7 µm). The mass spectrometer was operated in an information‐dependent acquisition mode. Data pretreatment, including peak detection and retention time correction, was achieved by XCMS and CAMERA packages implemented in R language. The metabolite identification was based on in‐house MS2 database, Human Metabolome Database (HMDB, www.hmdb.ca), and METLIN metabolite database (metlin.scripps.edu). The null value of the data was filled with the minimum value, and then the metabolites with QC variance less than 0.2 were retained. Metabolomics data were analyzed by log processing to approximate normal distribution, and *t*‐test was used to screen candidates and adjust P (FDR) value less than 0.05 as one of the screening criteria. The other criterion was VIP >1 by OPLS‐DA of R package ropls (v1.20.0)^[^ ^79^ ^]^ after standardized data. Correlations between differential bacteria and metabolites were calculated using Spearman correlation, and heatmap were generated using the ComplexHeatmap (v2.4.3)^[^ ^80^ ^]^ R package. Metabolite source classification and metabolite enrichment analysis were performed using a database MetOrigin.^[^ ^81^ ^]^

### Cell Culture

The colon cancer cell lines SW620 and HCT116 were obtained from ATCC. All cells were grown in DMEM (Gibco BRL, Grand Island, NY) supplemented with 10% fetal bovine serum (Sigma‐Aldrich, St Louis, MO).

### LPC Treatment, l‐Arginine Treatment, Cell Proliferation, and Cell Cycle Analysis

LPC and l‐arginine were obtained from Avanti Polar Lipids (Merck, Darmstadt, Germany). CRC cell lines HCT116 and SW620 were seeded at 5000 cells per well in a 96‐well plate. Cells were treated with vehicle (blank control), 10 µmol L^−1^ Inosine (negative control) or 20 µmol L^−1^ LPC, 20 µmol L^−1^ inosine, or 30 µmol L^−1^ l‐arginine respectively in DMEM supplemented with 10% fetal bovine serum for up to 3 days. For cell counting, cells were trypsinized and the number of cells was counted every day.

### Microbial DNA Extraction and Specific Bacteria Quantification

Mice fecal DNA was extracted using the DNeasy PowerSoil Kit (QIAGEN) according to the manufacturer's instructions. qPCR was performed to detect the *A. muciniphila* or *P. goldsteinii* level by using 20 ng genomic DNA in 20 µL universal SYBR Green PCR Master Mix (Takara) on the ABI QuantStudio 7 Flex Real‐Time PCR System. Specific bacteria quantitation was measured relative to the universal 16s gene. The primers used for detecting specific bacteria are listed (*A. muciniphila* forward primer: 5′‐GAGACGGCTA ACTCTGTGCC‐3′, reverse primer: 5′‐ GTTCATTACATGTCAAG ‐3′; *P. goldsteinii* forward primer: 5′‐CCGGCGCACGGGTGAGT‐3′, reverse primer: 5′‐ CTCAGTTCCAATGTG ‐3′).

### Statistical Analysis

The numerical variables between two groups were compared using unpaired Student's *t* test or Mann–Whitney U test where appropriate. Comparisons of categorical variables between two groups were performed using chi‐square test or Fisher exact test. Repeated measurement data were analyzed using two‐way analysis of variance test. All statistical analyses were conducted using GraphPad Prism, version 7.0 (GraphPad, La Jolla, CA) or R package. Differences were considered significant with *P* values <0.05. Additional methods are provided in Supporting Information. In the public metagenome analyses, the identification method of predominant confounding factors is based on previous studies.^[^ ^82^ ^]^ Alpha diversity as measured with the number of observed species was computed in all cohorts. *P* values were computed using a two‐sided Wilcoxon test, while the overall *P* value (on top) was calculated using a two‐sided blocked Wilcoxon test in the “coin” R package.^[^ ^83^ ^]^ In addition, beta diversity was assessed based on Bray–Curtis distance; *p* values of beta diversity based on Bray–Curtis distance were calculated with PERMANOVA by 999 permutations between sex groups or cohorts. Significantly difference species were identified via MaAsLin2 between male and female with CRC, where “cohort” was treated as the random effects.^[^ ^84^ ^]^

## Conflict of Interest

The authors declare no conflict of interest.

## Author Contributions

L.W. and Y.T. contributed equally to this work. L.W., Y.T., L.C., and Z.C. conceived and designed the research; L.W., Y.T., X.P., Y.Z., L.C., S.Y., S.Z., K.Y., S.S., H.X., Z.Y. performed experiments; L.W., Y.T., X.P., and Y.Z. analyzed the data; L.W., Y.T., X.P., Y.Z., S.Z., F.Y., J.Z., M.G., X.L., and Z.C. wrote the manuscript. All authors read and approved the final manuscript.

## Supporting information

Supporting Information

## Data Availability

The data that support the findings of this study are available from the corresponding author upon reasonable request.
