# Supplementary material for: Male‐Biased Gut Microbiome and Metabolites Aggravate Colorectal Cancer Development
Source: Adv Sci (Weinh). 2023 Jul 3;10(25):2206238. doi: 10.1002/advs.202206238 (PMC10477899; doi:10.1002/advs.202206238)
Supplement: Supplementary file 1 — Supporting Information [file ADVS-10-2206238-s001.pdf]

ADVANCED SCIENCE
Open Access

Supporting Information

for *Adv. Sci.*, DOI 10.1002/advs.202206238

Male-Biased Gut Microbiome and Metabolites Aggravate Colorectal Cancer Development

Ling Wang, Yi-Xuan Tu, Lu Chen, Yuan Zhang, Xue-Ling Pan, Shu-Qiao Yang, Shuai-Jie Zhang, Sheng-Hui Li, Ke-Chun Yu, Shuo Song, Hong-Li Xu, Zhu-Cheng Yin, Jun-Qiu Yue, Qian-Lin Ni, Tang Tang, Jiu-Liang Zhang, Min Guo, Shuai Zhang, Fan Yao, Xin-Jun Liang and Zhen-Xia Chen**

Supplementary Figures

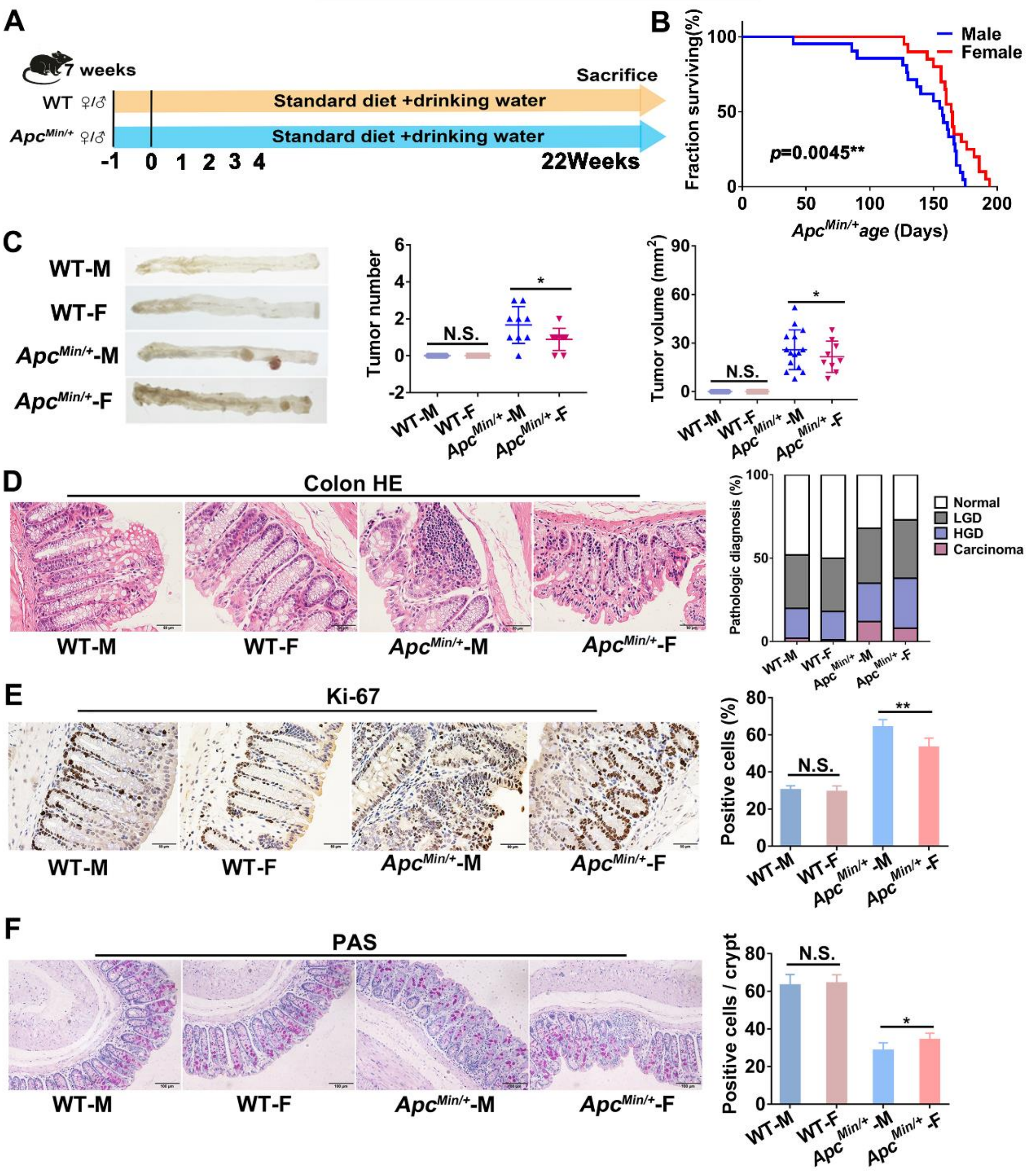

Supplementary Figure 1. Sexual dimorphisms in the tumorigenesis of *Apc*$^{Min/+}$ mouse model with standard diet. (A) Experimental design for an *Apc*$^{Min/+}$ CRC mouse model and WT mice with standard diet (n=20/each group). (B) Female had increased survival compared with male mice. (C) Representative image of colon at sacrifice. Tumor number and tumor volume in WT-male (WT-M), WT-female (WT-F), *Apc*$^{Min/+}$-male (*Apc*$^{Min/+}$-M), *Apc*$^{Min/+}$-female (*Apc*$^{Min/+}$-F) mice (n=9/each group). (D) HE staining for pathologic diagnosis of mice colons. Quantitative analysis of pathologic score was calculated according to the following criteria: 0, normal; 1, LGD;

2, HGD; and 3, carcinoma. (E) IHC staining for Ki-67 of mice colons with quantitative analysis of Ki-67 index. (F) The number of colon goblet cells was evaluated by PAS staining. The number of Paneth cells was evaluated by Lysozyme immunohistochemical staining. Six tissues were randomly selected from each section to calculate the percentage of positive cells per crypt. Data are expressed as mean ± SD. * $p < .05$, ** $p < .01$, N.S. no significant.

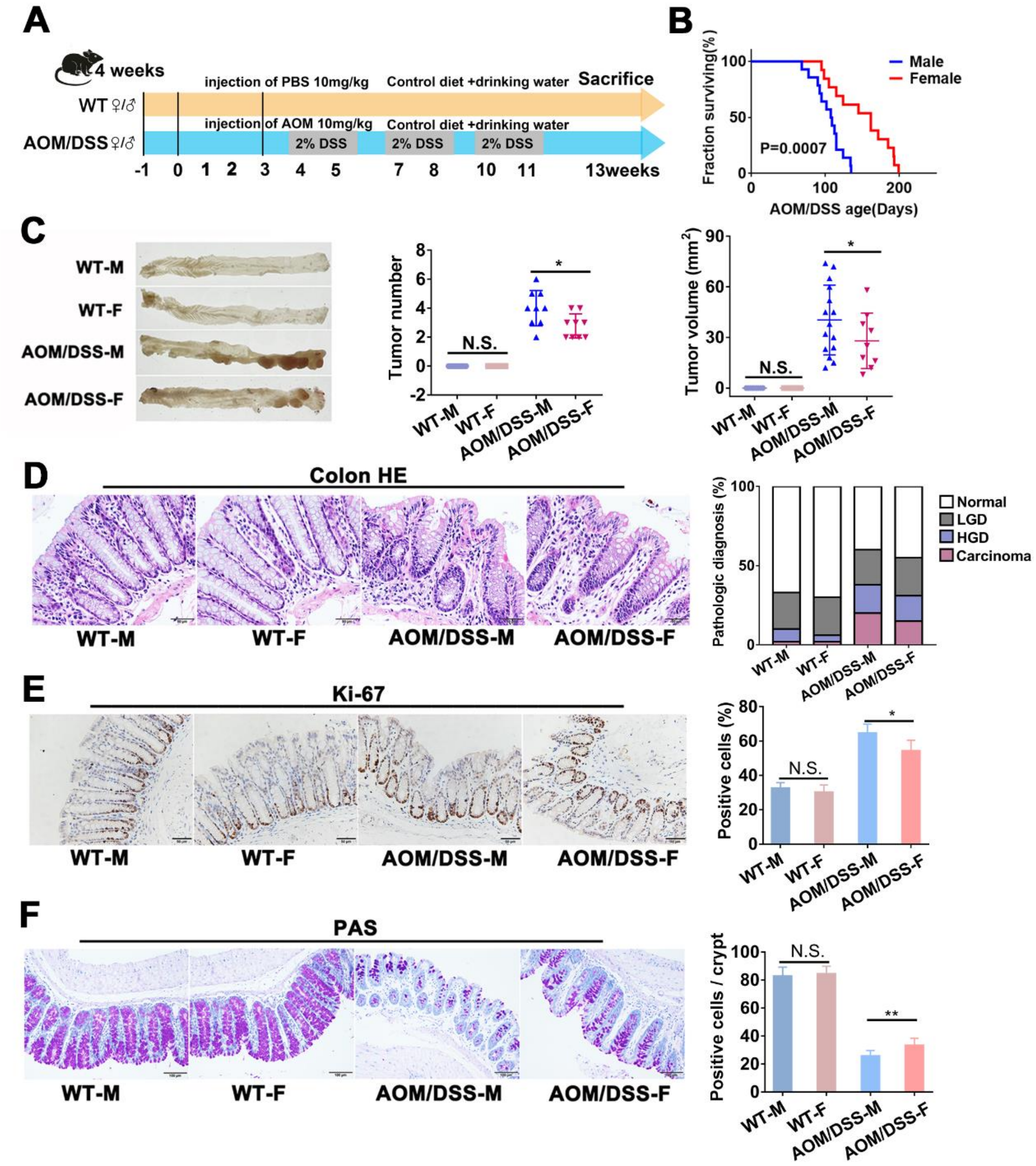

Supplementary Figure 2. Sexual dimorphisms in the tumorigenesis of AOM/DSS-treated mouse model. (A) Experimental design for an AOM/DSS-treated CRC mouse model and WT mice with standard diet (n=20/each group). (B) Female had increased survival compared with male mice. (C) Representative image of colon

at sacrifice. Tumor number and tumor volume in WT-male (WT-M), WT-female (WT-F), AOM/DSS-male (AOM/DSS-M), AOM/DSS-female (AOM/DSS-F) mice (n=20/each group). (D) HE staining for pathologic diagnosis of mice colons. Quantitative analysis of pathologic score was calculated according to the following criteria: 0, normal; 1, LGD; 2, HGD; and 3, carcinoma. (E) IHC staining for Ki-67 of mice colons with quantitative analysis of Ki-67 index in WT-M, WT-F, AOM/DSS-M, AOM/DSS-F mice. (F) The number of colon goblet cells was evaluated by PAS staining in WT-M, WT-F, AOM/DSS-M, AOM/DSS-F mice. Data are expressed as mean ± SD. * $p < .05$, ** $p < .01$, N.S. no significant.

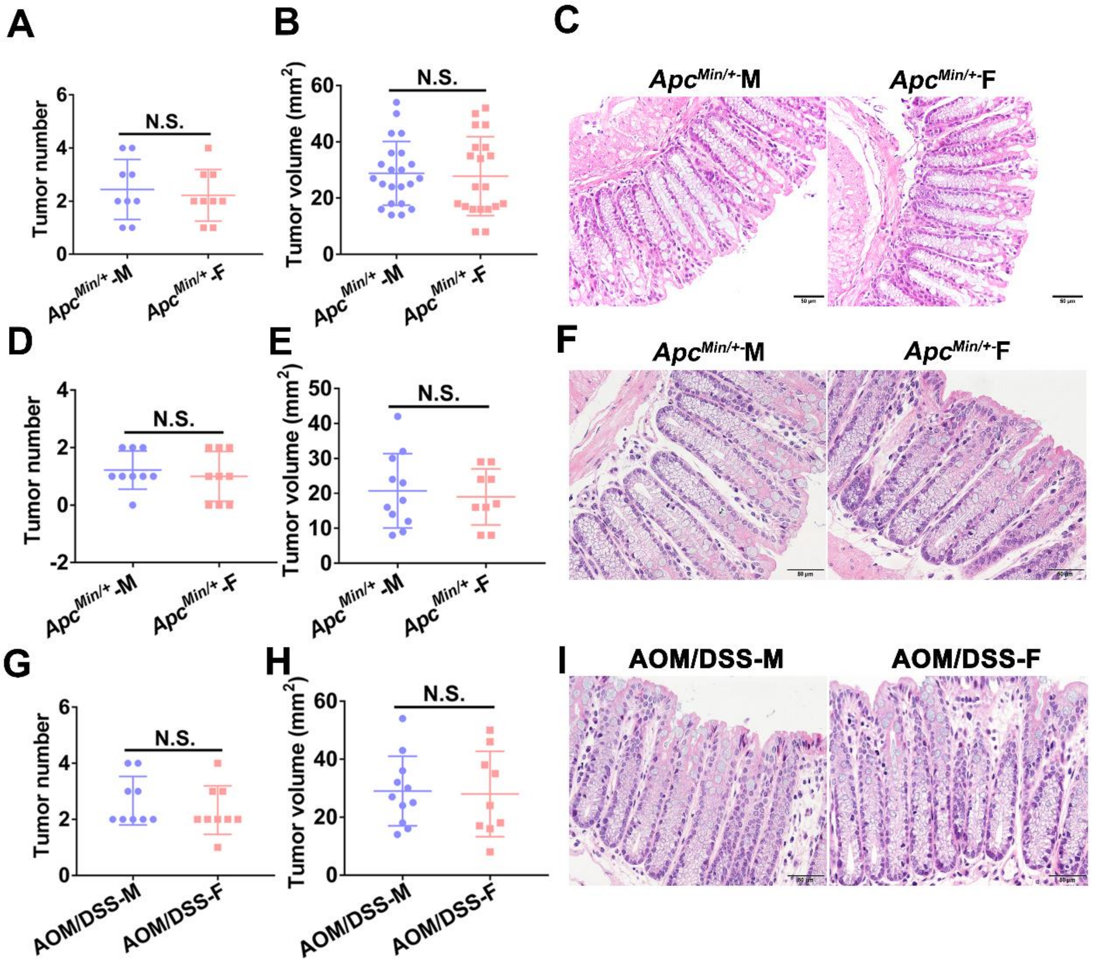

Supplementary Figure 3. Sex-biased gut microbiome contributes to sexual dimorphism in CRC development of mice. (A-B) Tumor number and tumor volume in male or female *Apc*$^{Min/+}$ mice with standard diet after antibiotic treatment (n=9/each group). (C) HE staining for pathologic diagnosis of mice colons in male or female *Apc*$^{Min/+}$ mice with standard diet after antibiotic treatment. (D-E) Tumor number and

tumor volume in male or female *Apc*$^{Min/+}$ mice with high-fat diet male or female *Apc*$^{Min/+}$ mice after antibiotic treatment. (F) HE staining for pathologic diagnosis of mice colons in male or female *Apc*$^{Min/+}$ mice with high-fat diet after antibiotic treatment. (G-H) Tumor number and tumor volume in male or female AOM/DSS-treated mice with standard diet. (I) HE staining for pathologic diagnosis of mice colons in male or female AOM/DSS mice after antibiotic treatment. Data are expressed as mean ± SD. N.S. no significant.

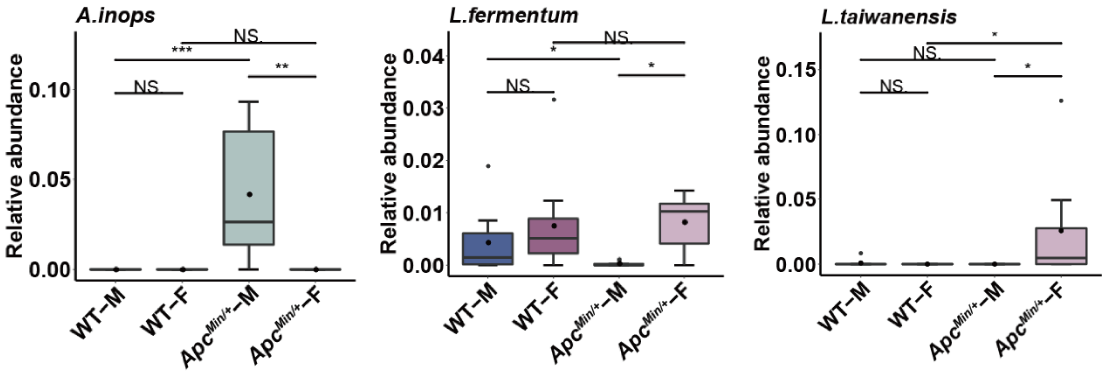

Supplementary Figure 4 (C) The abundance of *Lactobacillus taiwanensis, Lactobacillus fermentum* and *Alistipes inops* in WT-M, WT-F, *Apc*$^{Min/+}$-M, *Apc*$^{Min/+}$-F mice. Data are expressed as mean ± SD. * $p < .05$, ** $p < .01$. N.S. no significant.

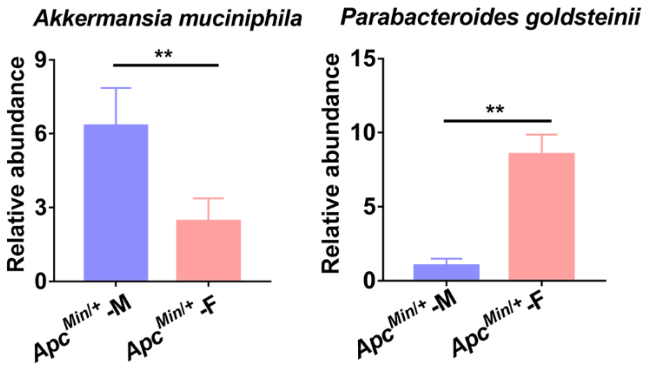

Supplementary Figure 5 The Relative abundance of *Akkermansia muciniphila* and *Parabacteroides goldsteinii* in *Apc*$^{Min/+}$-M and *Apc*$^{Min/+}$-F mice. Data are expressed as mean ± SD. * $p < .05$, ** $p < .01$.

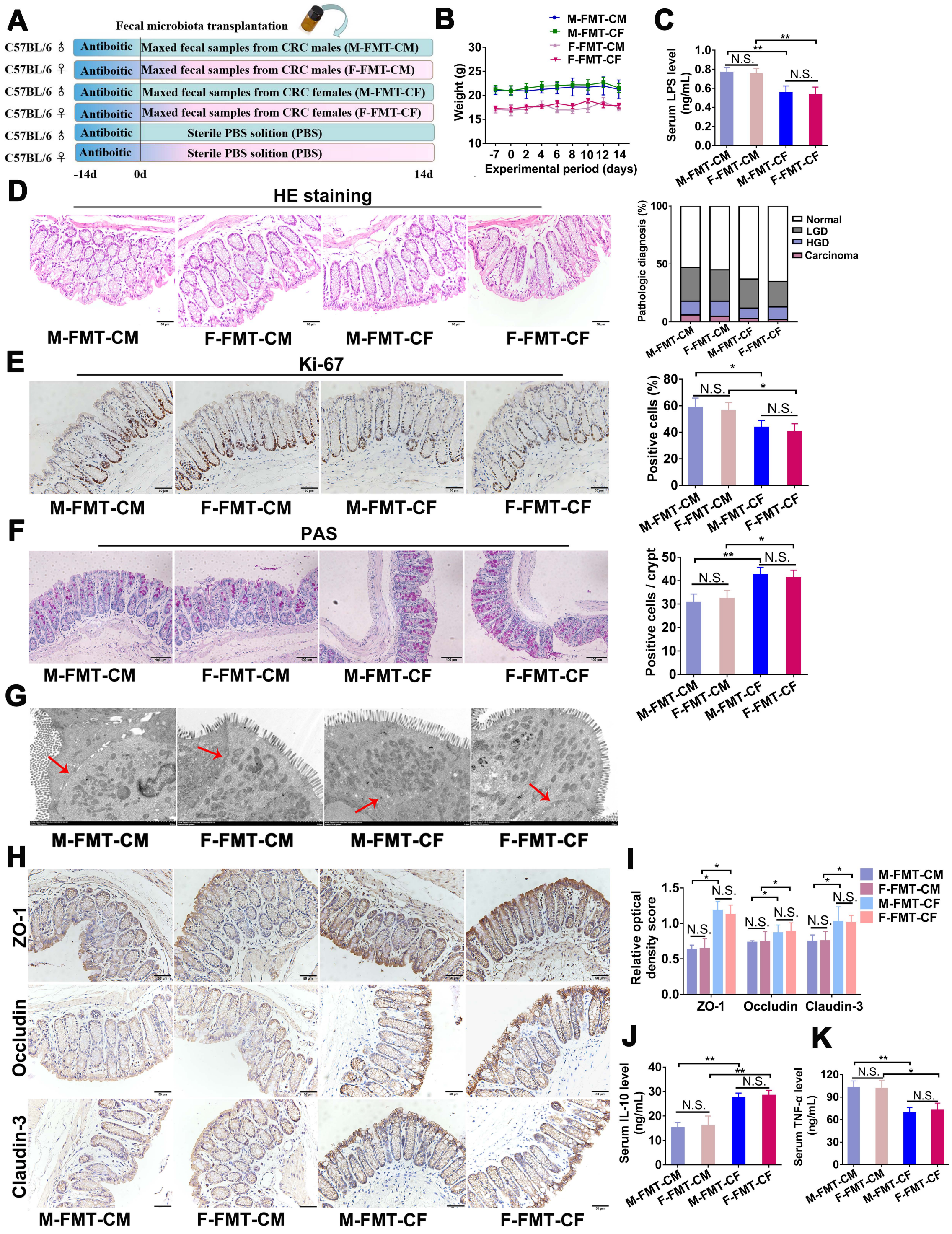

Supplementary Figure 6. Gut microbes from male CRC patients contribute to more severe colorectal tumorigenesis in recipient pseudo germ-free mice. (A) Experimental design for fecal microbiota transplantation for pseudo germ-free mice (n=15/each group). (B) Body weight of each group was recorded daily. (C) LPS concentration in serum of M-FMT-CM (male mice received feces from male patients), received feces from female patients), and F-FMT-CF (female mice received feces

from female patients) mice (n=9/each group). (D) HE staining for pathologic diagnosis of mice colons. Quantitative analysis of pathologic score was calculated according to the following criteria: 0, normal; 1, LGD; 2, HGD; and 3, carcinoma. (E) IHC staining of Ki-67 and quantitative analysis of Ki-67 index of colon section of pseudo germ-free mice. (F) The number of colon goblet cells was evaluated by PAS staining. The number of Paneth cells was evaluated by Lysozyme immunohistochemical staining. Six tissues were randomly selected from each section to calculate the percentage of positive cells per crypt. (G) Representative images of intercellular junctions of M-FMT-CM, F-FMT-CM, M-FMT-CF, and F-FMT-CF mice by transmission electron microscope. (H) IHC for distribution of the adhesion molecule ZO-1, Occludin and Claudin-3 with quantitative analysis in colon tissues of M-FMT-CM, F-FMT-CM, M-FMT-CF, and F-FMT-CF mice. (I) IL-10 concentration in serum of pseudo germ-free mice. (J) TNF-α concentration in serum of pseudo germ-free mice. Data are expressed as mean ± SD. * $p < .05$, ** $p < .01$, N.S. no significant.

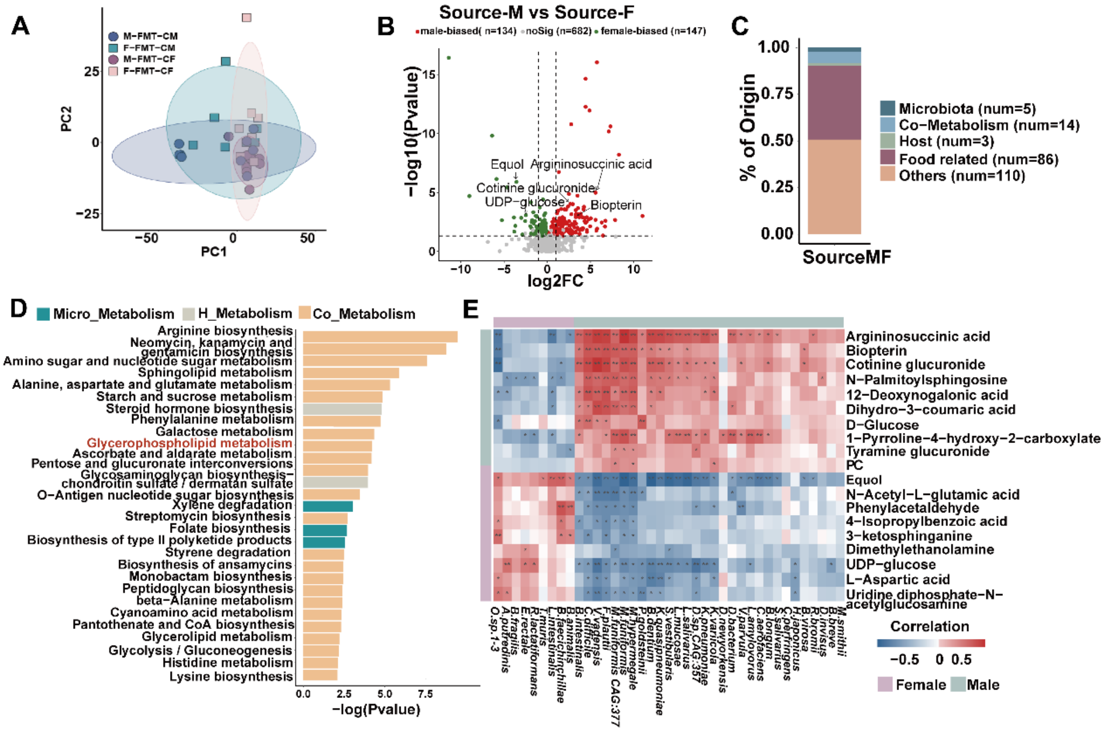

Supplementary Figure 7. Male-biased gut metabolites aggravated colorectal tumorigenesis through the glycerophospholipid metabolism pathway. (A) PCA plot for gut metabolomics analysis of M-FMT-CM (n=10), F-FMT-CM (n=10),

M-FMT-CF (n=9), and F-FMT-CF (n=9) mice. (B) Volcano plot for differential metabolites in comparison between source-M (mice received feces from male patients) and source-F (mice received feces from female patients) mice. (C) The source of differential metabolites among selected candidates. (D) Pathway analysis of differentially enriched metabolites among FMT-CM and FMT-CF mice. (E) Correlation analysis of the association of the sex-biased microbes and metabolites between FMT-CM and FMT-CF mice. Data are expressed as mean ± SD. * $p < .05$, ** $p < .01$, *** $p < .001$, N.S. no significant. Dot plot reflects data points from independent experiment.

Table S1. Clinical information of the human donors for stool gavage to mice.

ID	Sex	Age	BMI (kg/m2)	Weight (kg)	Height (cm)	TNM stage	Site of tumor	Smoking	Drinking
1	female	40	19.93	48.5	156	T3pN1M1; IV	sigmoid colon; liver	No	No
2	female	42	24.84	62	158	T3N1M0; IIIb	rectum	No	No
3	female	51	25.71	70	165	T4N2M1; IV	rectum; ovarian	No	No
4	female	51	25.64	64	158	T3N+M1; IV	sigmoid colon; liver	No	No
5	female	53	21.93	52	154	T4N1M1; IV	ileum; cecum	No	No
6	female	57	16.65	40	155	T3N0M1	colon; liver	No	No
7	female	57	16.44	47.5	170	TxNxM1; IV	rectum	No	No
8	female	57	30.00	73	156	T4N3M1; IV	colon; liver	No	No
9	female	59	23.01	56	156	T3N2M0; IIIB	colon	No	No
10	female	67	15.63	40	160	T2N0M0	rectum	No	No
11	female	68	16.65	40	155	T3N0M1	colon; liver metastases	No	No
12	female	69	21.08	50	154	T4bN0M1	rectum; liver metastases	No	No
13	female	71	26.84	62	152	T3N2M1; IV	rectum	No	No
14	male	31	19.94	59	172	T3N2M1	rectum; lung; liver	No	No
15	male	37	21.26	60	168	T4N1M1; IV	colon; pelvic metastasis	No	No
16	male	40	25.06	75	173	T3N0M0	right colon	No	No
17	male	45	22.86	70	175	TxNxM1	colon; liver	Yes	Yes
18	male	54	22.86	70	175	T3N2M1; IV	Left colon; liver metastasis	No	No
19	male	56	22.22	72	180	T4aN0M1	colon; lung; peritoneum metastasis	No	Yes
20	male	57	23.39	70	173	T3N0M1	rectum; liver	No	No
21	male	57	23.39	70	173	TxN+M1; IV	colon; liver	No	No
22	male	61	17.63	44	158	T3N1M1	colon; liver metastases	No	No
23	male	63	20.05	60	173	T3-4N+M1; IV	sigmoid; pelvic metastasis	No	No
24	male	63	20.07	58	170	IV	rectum; lung; liver	No	No

							metastasis		
25	male	63	18.42	52	168	T4N+M1; IV	colon; liver	Yes	Yes
26	male	64	23.66	70	172	T3N+M1	colon; liver	No	Yes
27	male	64	27.68	70	170	V	colon; lung; liver metastasis	No	No
28	male	72	20.44	57	167	T3N1M0; IIIb	colon	No	No
29	male	83	18.34	53	170	T3N1M1	rectum; liver metastases	No	No

Table S2. The differential microbes of *Apc*$^{Min/+}$ (male vs female)

Microbiome	pvalue	Log2FC
Dubosiella newyorkensis	0	-Inf
Flavonifractor plautii	0.032	-Inf
Clostridium symbiosum	0.032	-Inf
Eubacterium sp 14 2	0.032	-Inf
Clostridium methylpentosum	0.001	-Inf
Lactobacillus taiwanensis	0.032	-Inf
Collinsella massiliensis	0.032	-Inf
Leuconostoc lactis	0.032	-Inf
Parabacteroides goldsteinii	0.001	-12.87165254
Lactobacillus fermentum	0.016	-4.952644191
Anaerotruncus colihominis	0.002	-4.12655516
Akkermansia muciniphila	0.007	1.567886902
Clostridium sp ASF502	0.021	2.314882235
Romboutsia ilealis	0.038	2.645755528
Firmicutes bacterium ASF500	0.01	2.851567805
Fretibacterium fastidiosum	0.016	3.508204408
Lachnospiraceae bacterium 10 1	0.01	4.141943708
Lactobacillus johnsonii	0.001	7.572408754
Mucispirillum schaedleri	0.001	11.34133967
Muribaculaceae bacterium DSM 103720	0.001	Inf
Alistipes inops	0.001	Inf

Table S3. The differential metabolites from microbes of *Apc*$^{Min/+}$ (VIP >1 and FDR <0.05) (male vs female)

Metabolite	FDR	Log2FC
N-[(3a,5b,7b)-7-hydroxy-24-oxo-3-(sulfooxy) cholan-24-yl]-Glycine	0.000933482	5.971234163
Glycerophosphocholine	0.004117065	2.990826501
LysoPC (16:0)	0.000845617	2.876997937
Aldosterone 18-glucuronide	0.004117065	2.600221558
Dehydroisoandrosterone 3-glucuronide	0.004478912	2.373800634
PC (16:0/0:0)	0.000231737	2.116785368

Orotidine	0.000518124	2.064338348
Sulfolithocholylglycine	0.002103339	1.858640585
LysoPC (16:1(9Z))	0.000116533	1.82191341
Palmitoyl-L-carnitine	0.000146522	1.348601216
1-Stearoylglycerophosphocholine	0.000499737	1.020231054
N-Acetyl-D-glucosamine	0.016484495	0.670791441
Niacinamide	0.006447321	0.664620051
Pantothenic Acid	0.004117065	0.621987083
P-Tolualdehyde	0.007242809	0.364306864
Mesobilirubinogen	0.049074888	-0.725339033
6-(alpha-D-Glucosaminyl)-1D-myo-inositol	0.037442729	-0.790029277
N-Methylglutamic acid	0.008242076	-0.797607619
Alpha-Linolenic acid	0.029284833	-0.974523465
Phenylacetaldehyde	0.007242809	-1.666048452
16b-Hydroxyestrone	0.011294409	-1.857613225
4-Guanidinobutanoic acid	0.014097154	-2.062880835
L-Arginine	0.001101226	-2.908212203

Table S4. The differential metabolites (VIP >1 and P-value <0.05) between FMT-AM and FMT-AF

Metabolite	P-value	FDR	Log2FC
Oxoadipic acid	2.38E-04	8.53E-04	4.17E+00
Folinic acid	4.49E-05	2.41E-04	4.09E+00
Saccharopine	7.32E-07	7.87E-06	3.59E+00
Deoxycholic acid 3-glucuronide	5.50E-06	3.38E-05	3.32E+00
DTMP	3.43E-03	5.41E-03	2.87E+00
Porphobilinogen	1.29E-03	2.78E-03	2.72E+00
Homocysteine	2.94E-03	5.06E-03	2.26E+00
Biliverdin	7.04E-04	1.68E-03	1.96E+00
5-L-Glutamyl-L-alanine	6.76E-04	1.68E-03	1.84E+00
4-Hydroxycyclohexylcarboxylic acid	1.53E-03	3.14E-03	1.09E+00
Phenylacetylglutamine	6.79E-03	8.59E-03	9.05E-01
Hippuric acid	6.87E-04	1.68E-03	8.77E-01
Chlorogenic Acid	1.42E-02	1.59E-02	8.20E-01
4-Hydroxybenzaldehyde	8.17E-03	9.76E-03	6.21E-01
N-Acetyl-L-glutamic acid	7.12E-03	8.74E-03	6.09E-01
Cholic acid	2.15E-02	2.26E-02	5.12E-01
LysoPC(14:0/0:0)	3.95E-02	3.95E-02	3.30E-01
Mesobilirubinogen	4.45E-03	6.38E-03	5.39E-02
Dimethylethanolamine	2.30E-03	4.12E-03	-1.91E-01
2-Hydroxyphenylacetic Acid	1.92E-03	3.60E-03	-2.20E-01
Cuminaldehyde	1.43E-02	1.59E-02	-2.34E-01
Indole	3.62E-04	1.11E-03	-2.96E-01

5-(hydroxymethyl)-2-Furancarboxylic acid	1.35E-04	5.79E-04	-3.18E-01
P-Tolualdehyde	1.83E-04	7.13E-04	-3.60E-01
N-Acetyl-L-glutamate 5-semialdehyde	5.94E-03	8.21E-03	-4.89E-01
Androstenedione	6.11E-03	8.21E-03	-5.71E-01
3-ketosphinganine	3.16E-03	5.22E-03	-5.93E-01
PC(16:0/0:0)	3.93E-06	2.82E-05	-6.01E-01
LysoPC(16:0)	7.94E-09	1.71E-07	-7.89E-01
N-Acetylneuraminic acid	3.65E-03	5.41E-03	-8.65E-01
Dopaquinone	1.44E-02	1.59E-02	-8.70E-01
5-Hydroxyindoleacetaldehyde	3.02E-02	3.09E-02	-1.04E+00
D-Maltose	2.54E-06	2.19E-05	-1.15E+00
Ansamitocin P3	4.49E-04	1.29E-03	-1.16E+00
Indolelactic acid	6.59E-03	8.58E-03	-1.25E+00
Stachyose	9.92E-05	4.74E-04	-1.42E+00
Levan	7.06E-08	1.01E-06	-1.64E+00
Sinapic acid	2.60E-04	8.60E-04	-1.73E+00
Tetrahydrofolyl-[Glu](2)	1.62E-03	3.16E-03	-1.74E+00
Guanosine	1.54E-02	1.66E-02	-1.89E+00
Cytidine	3.55E-03	5.41E-03	-1.90E+00
7,8-Dihydropteroic acid	1.00E-03	2.26E-03	-2.32E+00
5-Aminoimidazole ribonucleotide	2.44E-10	1.05E-08	-4.59E+00

Table S5. The differential metabolites (VIP >1 and *P*-value <0.05) between FMT-CM and FMT-CF

Metabolite	P-value	FDR	Log2FC
Argininosuccinic acid	1.06E-05	6.80E-04	5.58E+00
12-Deoxynogalonic acid	1.26E-03	2.48E-02	3.71E+00
Biopterin	1.25E-03	2.48E-02	3.32E+00
PC	7.70E-03	6.12E-02	5.00E+00
N-Palmitoylsphingosine	7.11E-04	1.80E-02	2.79E+00
Cotinine glucuronide	9.45E-05	4.33E-03	2.68E+00
Tyramine glucuronide	1.33E-02	7.76E-02	2.66E+00
1-Pyrroline-4-hydroxy-2-carboxylate	1.35E-05	8.12E-04	2.51E+00
Dihydro-3-coumaric acid	5.90E-03	5.59E-02	1.51E+00
D-Glucose	3.90E-02	1.36E-01	1.18E+00
Dimethylethanolamine	1.80E-02	8.73E-02	-9.39E-02
4-Isopropylbenzoic acid	2.07E-02	9.40E-02	-4.09E-01

3-ketosphinganine	4.82E-02	1.57E-01	-4.18E-01
N-Acetyl-L-glutamic acid	1.78E-02	8.69E-02	-4.90E-01
L-Aspartic acid	1.47E-02	8.31E-02	-5.60E-01
Phenylacetaldehyde	4.30E-03	4.55E-02	-1.56E+00
Uridine diphosphate-N-acetylglucosamine	7.32E-03	5.97E-02	-1.96E+00
UDP-glucose	8.49E-04	2.04E-02	-2.47E+00
Equol	1.30E-06	9.63E-05	-3.56E+00
